# Sex-dependent effects of amyloid precursor-like protein 2 in the SOD1-G37R transgenic mouse model of MND

**DOI:** 10.1007/s00018-021-03924-5

**Published:** 2021-09-02

**Authors:** Phan H. Truong, Peter J. Crouch, James B. W. Hilton, Catriona A. McLean, Roberto Cappai, Giuseppe D. Ciccotosto

**Affiliations:** 1 Department of Biochemistry and Pharmacology, The University of Melbourne, Parkville, VIC 3010 Australia; 2 Anatomical Pathology, Alfred Hospital, Melbourne, VIC 3005 Australia; 3 Present Address: Oxidation Biology Unit, The Florey Institute of Neuroscience and Mental Health, The University of Melbourne, Melbourne, VIC 3010 Australia

**Keywords:** Amyloid precursor protein, Amyloid precursor-like protein, Motor neuron disease, SOD1-G37R, Sex differences, Amyotrophic lateral sclerosis

## Abstract

Motor neurone disease (MND) is a neurodegenerative disorder characterised by progressive destruction of motor neurons, muscle paralysis and death. The amyloid precursor protein (APP) is highly expressed in the central nervous system and has been shown to modulate disease outcomes in MND. APP is part of a gene family that includes the amyloid precursor-like protein 1 (APLP1) and 2 (APLP2) genes. In the present study, we investigated the role of APLP2 in MND through the examination of human spinal cord tissue and by crossing APLP2 knockout mice with the superoxide dismutase 1 (SOD1-G37R) transgenic mouse model of MND. We found the expression of APLP2 is elevated in the spinal cord from human cases of MND and that this feature of the human disease is reproduced in SOD1-G37R mice at the End-stage of their MND-like phenotype progression. APLP2 deletion in SOD1-G37R mice significantly delayed disease progression and increased the survival of female SOD1-G37R mice. Molecular and biochemical analysis showed female SOD1-G37R:APLP2−/− mice displayed improved innervation of the neuromuscular junction, ameliorated atrophy of muscle fibres with increased APP protein expression levels in the gastrocnemius muscle. These results indicate a sex-dependent role for APLP2 in mutant SOD1-mediated MND and further support the APP family as a potential target for further investigation into the cause and regulation of MND.

**Supplementary Information:**

The online version contains supplementary material available at 10.1007/s00018-021-03924-5.

## Background

Motor neurone disease (MND) is a fatal human neurodegenerative disorder, and the most common form of MND is amyotrophic lateral sclerosis (ALS) [1]. MND is characterised by the progressive destruction of motor neurons in the central nervous system (CNS) which causes muscle weakness, muscle atrophy, paralysis and ultimately death [2]. Despite progress in deciphering the molecular mechanisms of this disease, the cause and modulation of MND pathogenesis remains unclear. Whilst the sporadic causes triggering this disease account for the majority of patients diagnosed with MND, 5–10% of cases are inherited as familial MND [3].

MND patients display clinical heterogeneity with differences in the anatomical site of disease onset, symptom severity and rate of disease progression [4, 5] whilst age, sex, and various molecular targets are also contributing factors [6, 7]. This disease is predominantly presented in adulthood at around 55–70 years of age whilst a relatively small percent (~ 5%) of patients diagnosed with MND are around 20–30 years of age and in extremely rare cases, patients have been diagnosed before the age of 20 [8, 9]. Sex affects the incidence, clinical presentation and severity of the MND with men affected at 1.5-fold higher rate compared to women [5, 10]. However, this sex difference diminishes at post-menopausal ages [11] suggesting a protective role by estrogens in MND pathophysiology [7]. Sexual dimorphism in clinical phenotypes has been described in several studies, and they all consistently show a higher incidence of limb onset in men [12] whilst bulbar onset is more frequent in women [13, 14]. In rodent animal models, sex differences have been reported to occur in transgenic SOD1-G93A mice [15, 16] with the females displaying longer lifespans due to delayed symptom onset [17]. However, disparity in the literature exists, with some reporting that the onset of motor symptoms was detected earlier in male than in female SOD1-G93A mice [18]. The occurrence of sex differences in MND subjects makes sex comparison an important variable to be investigated in basic and preclinical research.

Several molecular factors have been identified that can modulate MND disease outcomes. One of these includes *Bax,* an important gene involved in the activation of the mitochondrial apoptosis pathway, where its deletion in the SOD1-G93A mice caused a delayed onset of disease symptoms and moderately increased its lifespan but failed to alter disease duration [19]. Similarly, the deletion of BH3-only protein, a pro-apoptotic protein important in regulating endoplasmic reticulum stress, in SOD1-G93A mice, improved motor neuron survival, delayed symptom onset and motor dysfunction, but failed to improve the lifespan in these mice too [20]. In contrast, the deletion of the PrP gene in SOD1-G93A mice significantly accelerated disease progression resulting in reduced lifespan [21]. Amongst these molecular modifiers, the amyloid precursor protein (APP), best known for its involvement in Alzheimer's disease pathogenesis, has been shown to modulate disease progression in SOD1-G93A mice [22]. Apart from being a genetic modifier, APP expression is associated with many physiological and pathological roles in the CNS and peripheral nervous systems (PNS). For example, following traumatic brain injury (TBI), APP protein expression levels are increased at the site of impact as part of a neuroprotective response [23]. APP expression is also upregulated in motor neurons undergoing programmed cell death as well as in ageing and injured neurons [24, 25]. Similarly, APP protein levels are upregulated in post-mortem spinal cord and muscle tissues in MND patients [26, 27] and in the rodent model for MND (SOD1-G93A mouse) at symptomatic age [26–28]. Moreover, a pilot study of MND patients revealed elevated levels of soluble APP cleavage products with a shift towards the non-amyloidogenic pathway of APP processing [29]. Regulating APP expression may have beneficial effects in MND since genetic deletion of APP (APP−/−) in the SOD1-G93A mice significantly slowed disease progression and promoted motor neuron survival, whilst the neuromuscular junction (NMJ) innervations in SOD1-G37R:APP−/− mice showed reduced denervation and improved muscle contractility [22]. Taken together, these observations indicate the potential involvement of APP in modulating disease progression in MND.

APP is part of a gene family that includes the amyloid precursor-like protein 1 (APLP1) and amyloid precursor-like protein 2 (APLP2) genes. Like APP, APLP2 also possess several important physiological roles that have been characterised in both the CNS and PNS. These include synaptogenesis [30], neurite outgrowth [31], axonal myelination [32], cellular adhesion and signalling [33], neuronal differentiation [34, 35], glucose and insulin homeostasis [36], brain metal homeostasis [37, 38], refractive development [39] and retinal development [40, 41] just to name a few. Therefore, to better understand if modulation of MND extends to other members of the APP gene family, we investigated the role of APLP2 in the SOD1-G37R transgenic mouse model. To do this, we examined the protein expression profile of both APLP2 and APP in human spinal cord samples from MND subjects and during disease progression in the SOD1-G37R mouse model. We then crossed APLP2 knockout (APLP2−/−) mouse with the SOD1-G37R mouse to generate SOD1-G37R mice expressing either two, one or no APLP2 alleles: SOD1-G37R:APLP2+/+, SOD1-G37R:APLP2 ± and SOD1-G37R:APLP2−/−, respectively, to determine if APLP2 expression affected disease progression by measuring motor function and motor neuron and muscle pathology. We also investigated this in both female and male lines to establish if any effects occurred in a sex-dependent manner.

## Materials and methods

### Human spinal cord tissue processing

Frozen sections of lumbar spinal cord were obtained from the Victorian Brain Bank (Australia) and the Multiple Sclerosis (MS) Society Tissue Bank (UK). Tissue samples were stored at − 80 °C until processed for analysis. Procedures involving handling of post-mortem human tissue were approved by a University of Melbourne Human Ethics Committee (Project ID 1238124). Briefly, human spinal cord tissue samples were homogenised in a tris(hydroxymethyl)-aminomethane-buffered saline (TBS)-based homogenisation buffer and the TBS-insoluble material was collected by centrifugation (21,000*g*, 4 °C, 15 min). This TBS-insoluble material was then resuspended in TBS-based homogenisation buffer supplemented with 1% (v/v) triton X-100 detergent and the samples centrifuged (18,000*g*, 4 °C, 5 min) to produce Triton X-100 soluble protein extracts which were assessed for protein content using the Pierce BCA Protein Assay kit, and the protein concentrations across the samples were normalised to a consistent protein concentration by diluting with the Triton X-100 supplemented homogenisation buffer. 10 µg protein samples separated by gel electrophoresis were performed as previously described [42].

### Mouse breeding

All mouse experiments in this study complied with the National Health and Medical Research Council code for the care and use of animals for scientific purposes and were approved by the University of Melbourne Animal Ethics Committee (Project Number: 1413304). Mice were housed on a reverse 12/12 hour light/dark cycle with access to feed and water ad libitum. The experimenter was blinded to age and genotype until completion of the behavioural testing. The SOD1-G37R, line 42, stock # 008342 [43], were sourced from The Jackson Laboratory (Bar Harbor, USA), are on a C57BL/6J background and are hemizygous for the SOD1-G37R transgene. The SOD1-G37R mouse colony was maintained by breeding the hemizygous SOD1-G37R mouse with a non-transgenic C57BL/6J (wild-type, WT) and identifying the hemizygous SOD1-G37R mouse by PCR genotyping. For the SOD1-G37R:APLP2−/− mouse line, the F1 breeding strategy involved mating the SOD1-G37R mouse with a APLP2−/− mouse to generate the WT:APLP2 ± and SOD1-G37R:APLP2 ± progeny at a ratio of 1:1. The F2 breeding strategy involved mating WT:APLP2 ± and SOD1-G37R:APLP2 ± mouse to generate six possible genotypes of equal weighting (1) SOD1-G37R:APLP2+/+, (2) SOD1-G37R:APLP2 ± , (3) SOD1-G37R:APLP2−/−, (4) WT:APLP2+/+, (5) WT:APLP2 ± , and (6) WT:APLP2−/− at 1:6 ratios.

### Mouse monitoring and end-stage disease

Mouse body weights were recorded weekly and from 3 weeks of age (at weaning age), the health monitoring frequency was increased to three times per week and then to daily after 25 weeks of age as the MND symptoms became more progressive and severe. Mice were killed when they reached pre-symptomatic (12 weeks), symptomatic (22 weeks) and End-stage (~ 28 weeks) and similar numbers of control WT mice at similar ages were killed at the same timepoints for direct comparison. End-stage is defined when the mouse displayed one or more of the following criteria points; no longer able to perform the rotarod task, unable to right itself within 15 s of being placed on either side, when paralysis was observed in at least one hindlimb or if they lost 15% of their maximum recorded weight. The same number of WT:APLP2+/+ and WT:APLP2−/− were killed at the same time for age matching. Mice were killed by an intraperitoneal injection of a cocktail of xylazine (16 mg/kg body weight) and ketamine (120 mg/kg body weight) and followed by opening of the pneumothorax and transcardial perfusion with PBS solution.

### Mouse neurological scoring

For each mouse, a neurological score was calculated for both hindlimbs. The neurological scores were assigned using an amended scaling system of the neurological scoring system developed at the ALS Therapy Development Institute [44]. Briefly, a score of 0.5–1.0 was assigned when the onset motor symptoms, as defined by the mouse displaying tremoring of hind legs and partial collapse of the leg extensions from the lateral midline when suspended by its tail, a score of 1.5–2.0 was assigned when the mouse displayed a complete or partial collapse of hindlimbs, signs of hindlimb muscle atrophy or forelimb tremoring, a sign of toes curling during the tail suspension test or if they curled under at least twice during a 30 cm walk or if any part of a foot was dragging along the cage bottom/table), a score of 2.5–3.0 assigned when the mouse developed a very wobbly gait, prominent signs of muscle atrophy in the hindlimb, rigid paralysis or minimal joint movement, foot not being used for generating forward motion or unsteady when walking, and a score 3.5–4.0 assigned when they displayed rigid paralysis and no forward motion, paralysis in one or more limbs, or the mouse cannot right itself within 15 s after being placed on either side.

### Mouse rotarod performance

Locomotor function was assessed using the rotarod instrument (IITC Life Science, Woodland Hills, CA, USA) set to 4 rpm and increasing to 40 rpm over 180 s. Prior to testing day, mice were habituated to the instrument for two consecutive days and allowed to explore the apparatus set at a constant speed of 10 rpm for 300 s. Following habituation, mice were trained on the rotarod for 3 consecutive days with the rotation speed initially set to 4 rpm and increasing to 40 rpm over 180 s. Mice that were still on the rotarod after 180 s were recorded as having no detectable locomotor deficit. Mice that could not continue on the rotarod for the 180 s would fall safely on to a padded base. The fall latency was recorded by the experimenter and is defined as the physical fall off the rotarod or if their hindlimbs slip off and they use their front paws remain grasping to the rotarod. Rotarod performances were performed and recorded 2 days per week, with three trials on each day of testing and intervals of 10 min of rest provided between each test trial. The mice were tested from 8 weeks of age up until the disease End-stage and matching time points in control mice.

### Mouse DigiGait analysis

Analysis of the mice’s gait was conducted using the DigiGait instrument and analysis performed using the supplied software analysis (Mouse Specifics, Inc, USA). Prior to testing the mice on this instrument, they undergo a pre-training period for 3 days to acclimatise to the instrument and a range of treadmill speeds were tested to determine the maximum speed the mice could tolerate safely. After the training period, DigiGait test recordings were taken for each mouse weekly from 7 weeks of age. Mice were placed on the DigiGait treadmill and setting the belt speed at either 10, 15, or 20 cm/s and a 15 s recording taken.

### Mouse tissue collection

Mice were killed and tissues were collected when SOD1-G37R mice reached pre-symptomatic (12 weeks), symptomatic (22 weeks) and End-stage (~ 28 weeks) and a similar number of control WT mice of similar ages were killed at the same timepoints for direct comparison. For biochemical analysis, the brain, spinal cord, and hindlimb skeletal muscle organs were dissected free, immediately snap frozen in liquid nitrogen and then transferred to clean tubes for storage at − 80 °C until processing. For histological analysis, the spinal cord tissue was carefully removed from the spinal cord canal and the lumbar region identified and dissected free and postfixed in freshly prepared 4% paraformaldehyde in PBS solution overnight then cryo-protected by incubation in a 30% sucrose solution (v/v in PBS) for at least 24 h. The harvested lumber spinal cord or hindlimb skeletal muscle (which were laid flat on the end of a syringe) were embedded in Optimal Cutting Temperature compound (Tissue-Tek; Sakura FineTek) and snap frozen in a bath of isopentane pre-chilled in liquid nitrogen and then wrapped in plastic and stored at − 80 °C until cutting for histochemistry.

### Mouse tissue processing for biochemical analysis

Brain and spinal cord tissue samples were weighed and lysed at 1:5 w/v in brain lysis buffer (50 mM Tris–HCl, 150 mM NaCl, 0.1% TritonX-100, pH 7.5) by passaging the tissue several times through a series of 18G and 22G needles followed by a homogenization step using a chilled handheld homogenizer that was kept on ice during the procedure. Harvested muscle samples were weighed, minced into very small pieces using a clean sterile scalpel blade whilst on a Petri dish resting on ice, then transferred to a centrifuge tube and muscle lysis buffer added to 1:5 w/v (50 mM Tris–HCl, 150 mM NaCl, 0.1% TritonX-100, 0.1% SDS, 10 mM EDTA, 1 mM dithiothreitol). The tubes were placed in a chilled sonicating water bath and sonicated with three 30 s bursts. To complete homogenization, all homogenate samples were incubated on ice for 20 min then centrifuged at 15,870*g* for 30 min at 4 °C. The supernatant was transferred to a new tube and the protein concentration quantified using the bicinchoninic acid assay (Pierce, Rockford, IL, USA). Lysis buffer was added to the samples to normalise protein concentration across the samples to 2 mg/ml before gel electrophoresis. Proteins were resolved by SDS-PAGE electrophoresis under reducing and denaturing conditions by pre-mixing equivalent amounts of protein samples with 2X Tris–glycine SDS sample loading buffer (0.16 M Tris, 4% SDS, 20% glycerol, 0.04% bromophenol blue). Samples were heated at 95 °C for 5 min, allowed to cool for 5 min followed by a quick centrifugation step then loaded into 4–12% NuPAGE Bis–Tris pre-cast gel and the gels were run according to manufacturer’s instructions (Invitrogen, Australia). Resolved gels were transferred onto nitrocellulose membranes (BioRad, Australia) using the wet tank blotting system (BioRad, Australia).

### Western blot analysis

Briefly, the membranes were incubated in blocking buffer (5% skim milk in PBST (0.05% Tween-20 in PBS) for 1 h at room temperature and then probed using an anti-APP antibody 22C11 (APP 66–81) produced in house [45], anti-human SOD1 (Abcam, 1:10,000) and an anti-APLP2 95/11 a rabbit polyclonal antiserum raised against recombinant APLP2 (28–693) protein [46] and expressed in Pichia pastoris (as previously described) [47]. Loading control GAPDH was detected using Cell Signaling Technology antibody (#2118). Blots were incubated in primary antibody diluted in PBST overnight at 4°C. The next day, the membranes were washed in PBST buffer, incubated with a secondary antibody conjugated to horseradish peroxidase for 2 h at room temperature and washed in TBST. Immunoreactivity was detected using the enhanced chemiluminescence reagent (ECL-plus, GE Healthcare, UK) and imaged on a ChemiDoc digital imaging system (BioRad, Australia). Protein expression levels were quantitated by densitometry analysis of band intensities using Image J/Fiji software (ver. 1.52e, NIH). The intensity value for each immune-reactive band was normalised to its corresponding housekeeping loading control to account for variability in protein loading across samples.

### Immunohistochemistry of mouse spinal cord sections

Serological transverse tissue sections (1:10) of the lumbar spinal cord were cut at 20 µm thickness (serially) using a cryostat machine (Leica) and were collected and adhered to superfrost plus slides (Fisher Scientific). Immunohistochemistry was prepared by briefly washing sections with PBS buffer three times (5 mins per wash), permeabilised (0.3% Triton X in block buffer for 20 min) and blocked (10% goat serum in PBS) for 1 h. Tissue sections were incubated with primary antibodies to GFAP (Merck Millipore, 1:500), IBA1 (Wako, 1:500), APP (22C11, 1:50, produced in-house), and CHAT (Invitrogen, 1:500) diluted in blocking buffer overnight at 4 °C in a humidifier chamber. The next day, antibodies were removed and slides washed with PBS buffer (three times, 10 min each) before incubation in a secondary goat anti-mouse or goat anti-rabbit antibody (conjugated to horseradish peroxidase (HRP)) for 2 h at room temperature. Tissue sections were PBS washed (three times, 10 min each) then incubated in DAB enhancement solution (ImmPACT DAB peroxidase-HRP substrate, SK-4105, Vector labs, Australia). Tissue sections were rinsed with distilled water three times then dehydrated in a series of ethanol solutions, cleared in xylene solution two times and mounted in Safety mounting medium (Trajan, Grale). Spinal cord sections were imaged using a digital slide scanner (Panoramic SCAN II, 3Dhistech, Hungary) using a Carl Zeiss Plan Apochromat 20 × /NA 0.8 objective (Zeiss, Germany) and the acquired images of tissue sections were viewed using Case Viewer software (ver 2.2, 3Dhistech, Hungary).

### Nissl staining of mouse spinal cord tissue sections and neuron analysis

Nissl stains were performed to assess neurons in the lumbar sections of the spinal cord. 20-µm thick frozen tissue sections were air dried at RT for 1 h on glass slides, washed in PBS then soaked in 1:1 v/v alcohol/chloroform solution overnight, then rehydrated in a series of ethanol solutions (100%, 90% and 70%) and distilled water. Slides were stained with 0.1% cresyl violet solution (0.1 g cresyl violet acetate, 100 ml distilled water, 0.3 ml glacial acetic acid) for 1 h in a 37 °C oven. The slides were rinsed in distilled water for 3 s and dehydrated in a series of ethanol solutions (70%, 90% and 100%), cleared in xylene solution two times (5 min each) and mounted in Safety mounting medium (Trajan, Grale). The spinal cord sections were imaged using a digital slide scanner Panoramic SCAN II machine using a Carl Zeiss Plan Apochromat 20 × /NA 0.8 objective (Zeiss, Germany). The acquired images were viewed using Case Viewer software (ver 2.2, 3Dhistech, Hungary). For each Nissl stained spinal cord section, the left and right ventral horn regions were selected as regions of interest (ROIs). Neurons were analysed using the manual thresholding command, followed by cell segmentation and particle analysis with neurons having a soma diameter of less than 10 µm excluded from further analysis. A total of seven spinal cord sections were analysed for each animal and the distance between each serial section analysed was at least 200 µm apart.

### Neuromuscular junction assessment in mouse tissue

Serological longitudinal sections (1:5) of TA muscle cut at 10 µm thick were collected on superfrost plus slides (Fisher Scientific). Muscle sections prepared for immunofluorescence were fixed in 4% PFA for 1 h and briefly washed with PBS buffer three times (5 min per wash). All tissue sections were incubated with permeabilisation buffer (0.3% TritonX-100, 10% goat serum in PBS) for 60 min, followed by blocking buffer (10% goat serum in PBS) for 60 min and then in primary antibody diluted in blocking buffer (Synaptophysin, Santa Cruz, 1:500)) in a humidifier chamber overnight whilst at 4 °C. The following day, diluted antibody was removed, sections washed with PBS buffer three times (10 min per wash) then incubated in a fluoro-tagged secondary antibody (goat anti-mouse or goat anti-rabbit) prepared in blocking buffer and containing DAPI (2 µg/ml) for 1 h at room temperature and in the dark. Neuromuscular junctions (NMJs) were visualised by adding FITC-alpha-bungarotoxin (CFTM 488A, Biotium, Australia) at 1 µg/ml diluted in blocking buffer solution. All tissue sections were washed with PBS (three times, 10 min each) and then covered with antifade mounting medium (Prolong Gold, Invitrogen) and mounted with a glass coverslip. The slides were air dried at room temperature for at least 24 h in the dark before imaging. The stained tissue sections were visualised through a 20X objective using a Zeiss Axioplan 2 microscope and images taken with a Coolscope snap camera and Zen 2 software (Zeiss, Germany). The same exposure settings were used for all mice genotypes and exported in a TIF format, and the fluorescence intensity levels quantified using Image J/Fiji software (ver. 1.52e, NIH).

The innervation status of each NMJ was assessed by determining the level of co-localisation between the muscle fibre end plate (α-bungarotoxin, green) and axon terminal of motor neuron (synaptophysin, red). Colocalisation levels were measured using Imaris software (ver. 9.1, Bitplane, USA) and greater than 150 NMJs were examined in each animal for analysis. For each NMJ, two separate surfaces were created for each channel to allow the creation of a co-localisation surface containing the overlapped region of the two surfaces. The volume of the three surfaces was used to calculate the percentage of colocalization or NMJ innervation.

### ATPase staining for mouse muscle tissue

The muscle fibre types in the gastrocnemius (GA) muscle located in the mouse hindlimb were analysed by histochemical staining for myosin ATPase activity based on a previously published protocol [48]. Briefly, serological transverse sections (1:10) of the GA muscle was cut at 20 µm thickness and adhered to Superfrost plus glass slides (Fisher Scientific) by incubation at room temperature for 1 h. Slides were washed in PBS then fixed in 4% paraformaldehyde solution (prepared in PBS) for 1 h and then washed with PBS three times. Two sets of muscle slides were incubated in Myosin ATPase activity buffer with 1 slide set incubated in buffer at pH 4.3 and the second slide incubated in a buffer set at pH 10.2. For Myosin ATPase pH 4.3 treatment, slides were pre-incubated in 0.1 M acetate buffer at pH 4.3 for 10 min and for Myosin ATPase pH 10.2, slides were pre-incubated in Baker’s Formal Calcium solution for 1 min. Slides from both sets were then briefly washed in distilled water and placed in sodium barbital solution and incubated at 37 °C for 30 min. Slides were brought to room temperature and washed in distilled water three times, incubated in fresh 1% CaCl_2_ for 10 min followed by incubation in 2% CoCl_2_ for 10 min. Slides were washed thoroughly in distilled water and the colour allowed to develop for 15 s in freshly prepared 1% ammonium sulphide solution in a fume cupboard and then slides were washed with distilled water. Slides were dehydrated through the series of 70%, 90% and 100% alcohol solutions, cleared with xylene and finally mounted in safety mounting medium (Trajan, Grale) with a glass coverslip.

### Mouse muscle fibre typing analysis

Stained muscle sections were imaged using a Zeiss Axioscope 2 light microscope through a 10 × objective and images were acquired using Axiocam 503 colour camera with Zenpro software 2011 (Zeiss, Germany). Acquired images were exported in TIF format and imported into Image J/Fiji software (ver. 1.52e, NIH) for further analysis. Total fibre numbers and the composition of each fibre type group were counted manually using the cell counter function. For each muscle fibre type, the cross-sectional area of each muscle fibre cell was measured using ROI function. Over 150 muscle cells were measured per animal.

### Statistical analysis

All data are expressed as mean ± SEM with *p* values of 0.05 or less considered as significant. A student’s *t* test was used to compare between two experimental groups. Multiple comparisons were assessed using a one-way analysis of variance with Bonferroni’s post hoc test to compare different genotypes within the same sex groups. A two-way analysis of variances was used when assessing between different genotypes, age and sex, followed by Tukey’s post hoc tests. To determine the time in which mice showed a reduction in their rotarod performance, a split line regression model using GenStat (ver. 6, VSN International, UK) analysis package was used to fit the performance curve for each animal followed by student’s *t* test using GraphPad Prism software (Ver. 7, San Diego, CA, USA) to compare between two sex groups. All other statistical analyses were performed using GraphPad Prism software.

## Results

### APLP2 and APP protein expression are upregulated in the human MND spinal cord in a sex-dependent manner

**Fig. 1:**
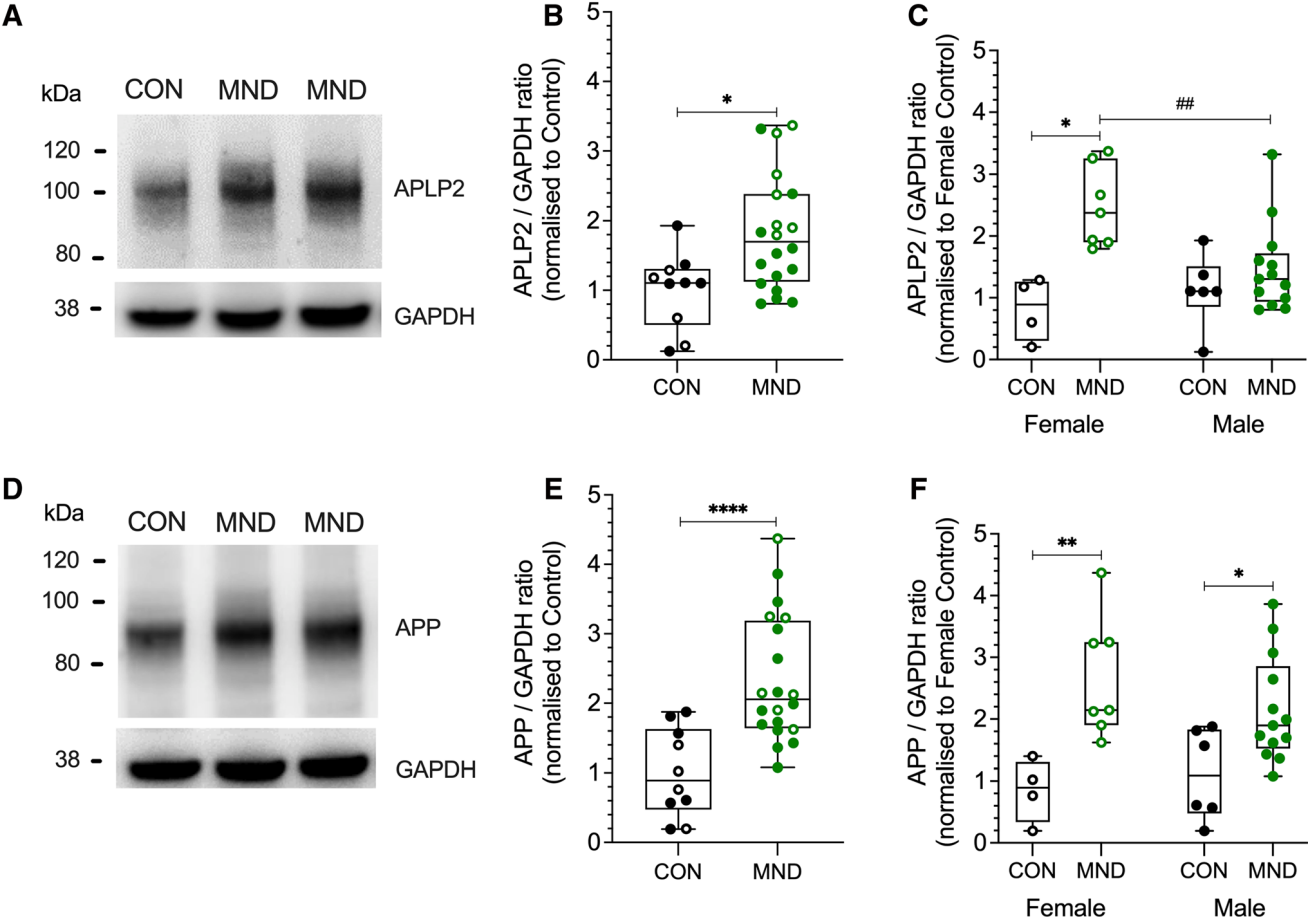
APLP2 and APP protein expression is increased in human, MND-affected spinal cord. Human spinal cord tissue samples from MND (green) and healthy age-matched control (CON) patients (black) were analysed by Western blot and densitometric quantification performed for **A–C** APLP2 and **D–F** APP protein. Data presented for **B, E** combined sexes and for **C, F** female (open symbol) and male (filled symbol) sexes separately. All APLP2 and APP data were expressed as ratio of GAPDH (loading control) and protein expression levels for MND samples normalised relative to control patients of **B, E** combined sexes and **C, F** female only cohort. Data are presented in a box and whiskers plot, median, minimum and maximum values of individual data points are shown. Statistical significance was determined by Mann–Whitney unpaired student *t* test for plots (**B, E)** and a two-way ANOVA with Bonferroni multiple comparison testing for plots (**C, F)**, *p < 0.05, **p < 0.01, and ****p < 0.0001, and female versus male, ^##^*p* < 0.01. [*n* = 4/6 and 8/12 (M/F) for CON and MND, respectively]

We assessed the protein expression levels of APLP2 and APP proteins in post-mortem spinal cord samples from human MND cases and compared them to age-matched healthy control samples (Table 1, Fig. 1). The APLP2 protein expression level in the MND patient group was 1.8-fold higher and significantly different compared to the control group (Fig. 1A, B). To determine whether a sex-based difference was evident in these patient samples, we compared the female and male groups separately. We observed APLP2 protein expression was at least 2.4-fold higher in the female MND group and that this was significantly different to both the control female and to the MND male patients (Fig. 1C). Like APLP2, the APP protein expression levels are congruent with those previously reported [26, 27] showing a 2.3-fold higher protein expression of APP in the MND-affected spinal cord samples and significantly different to the control group (Fig. 1D, E). In contrast to APLP2 results, APP levels were significantly elevated in MND patient samples in both female and male groups by 2.6- and 2.1-fold compared to control groups, respectively, but no differences were observed between female and male groups (Fig. 1F).

**Table 1:** Demographic information of spinal cord samples for control and MND cases

Disease group	Mean		
	Sex (male:female)	Age (years)	Post-mortem interval (hours)
Control	1.8:1	79.4 (13.1)	37.3 (25.5)
MND	2.4:1	67.1 (9.0)	34.0 (16.4)

Mean (standard deviation)

### APLP2 and APP protein expression are upregulated at End-stage disease in a sex-dependent manner in the SOD1-G37R mouse

**Fig. 2:**
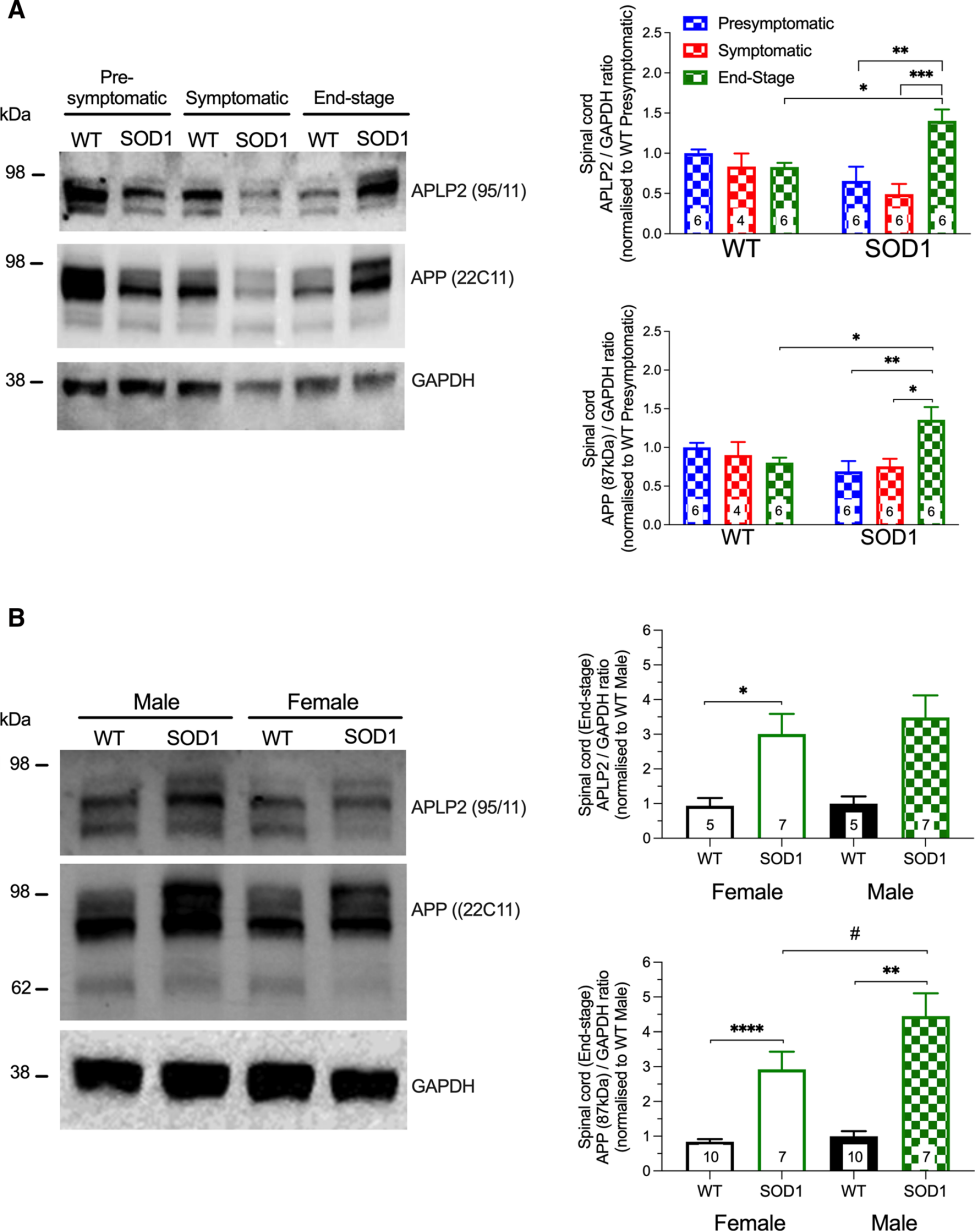
Elevation of APLP2 and APP levels in the spinal cord of SOD1-G37R during disease progression. Spinal cord lysis samples from WT and SOD1-G37R mice (SOD1) at pre-symptomatic (12 weeks, blue), symptomatic (22 weeks, red) and End-stage (28 weeks, green) diseases were analysed by **A** Western blot and protein expression of APLP2 (antibody 95/11), APP (antibody 22C11), and GAPDH (loading control) was determined. Blots were imaged and densitometric analysis of protein bands was expressed as a ratio of GAPDH levels normalised to the WT pre-symptomatic group. **B** Spinal cord samples of female and male mice from End-stage SOD1 (green) and WT (black) samples were analysed by Western blot analysis for APLP2 and APP immunoreactivity, densitometric analysis performed, protein band levels expressed as a ratio of GAPDH levels and then normalised to the WT male group. Data presented as mean ± SEM. Statistical analysis performed using two-way ANOVA with Bonferroni’s post hoc test *p < 0.05, **p < 0.01, ***p < 0.001, ****p < 0.0001 and ^#^*p* < 0.05 for female versus male groups. *n* = 4–10

We next assessed the endogenous protein expression level of APLP2 and APP by Western blot analysis in the SOD1-G37R mouse at different disease progression time points and compared the results to the age-matched WT littermate controls. Tissue lysates were prepared from organs of the SOD1-G37R mouse model that are most affected by MND; the spinal cord, brain stem, cerebellum and hindlimb muscle. These organs were harvested at three well-characterised disease time points termed pre-symptomatic (12 weeks), symptomatic (22 weeks) and End-stage (~ 28 weeks). In the spinal cord, both APLP2 and APP protein expression levels were significantly elevated in the SOD1-G37R mouse at End-stage (*p* < 0.05) compared to the age-matched WT control mouse (Fig. 2A) and demonstrating the recapitulation of this human disease phenotype. When comparing the three disease progression time points in the SOD1-G37R mouse, both APLP2 and APP protein expression levels in the spinal cord were significantly higher at End-stage compared to both the pre-symptomatic and symptomatic stages (*p* < 0.01, Fig. 2A). In contrast, similar protein expression levels of APLP2 and APP were measured in the brainstem, cerebellum and hindlimb muscle and they were also similar when compared across the different disease progression time points in the SOD1-G37R mouse and again when compared to the age-matched WT control group (Figure S1).

Female and male mice were compared to determine whether the elevated levels of APLP2 and APP protein expression levels in the spinal cord of the SOD1-G37R mouse at End-stage was sex dependent. Whilst the APLP2 protein level was similarly upregulated in both sexes in the SOD1-G37R mouse at End-stage compared to the WT group, a statistical significance was only achieved for the male mouse (*p* < 0.05, Fig. 2B). The APP protein level was significantly upregulated in both sexes too in the SOD1-G37R mouse compared to respective WT littermate controls and it was also significantly higher in the male compared to the female SOD1-G37R mouse (*p* < 0.05, Fig. 2B). These results demonstrate that APP and APLP2 protein expression levels are significantly upregulated in the spinal cord at End-stage in SOD1-G37R mouse, an effect that is sex dependent.

### The lifespan is increased in female but not in male SOD1-G37R mouse lacking APLP2 gene expression

**Fig. 3:**
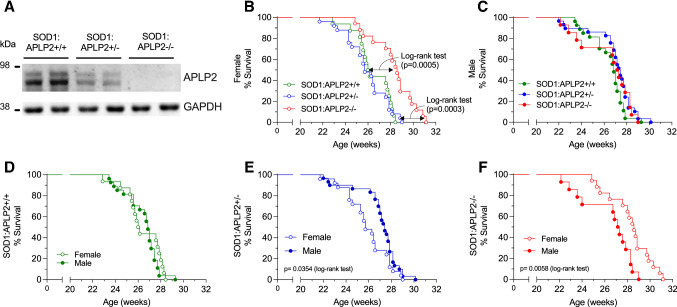
APLP2 deletion improves life span of female SOD1-G37R mice. **A** Western blot image of spinal cord tissue samples expressing APLP2 protein in crossbred mice for SOD1-G37R:APLP2+/+, SOD1-G37R:APLP2 ± and SOD1-G37R:APLP2−/− mice. Survival curves plotted for **B** female and **C** male for SOD1-G37R:APLP2+/+ (green), SOD1-G37R:APLP2 ± (blue) and SOD1-G37R:APLP2−/− (red) mice groups. Survival curves with log-rank (Mantel–Cox) test were compared between sexes for **D** SOD1-G37R:APLP2+/+, **E** SOD1-G37R:APLP2 ± and **F** SOD1-G37R:APLP2−/− mice. *n* = 14–27

To determine whether modulating the APLP2 expression would affect the limiting 28-week lifespan of the SOD1-G37R mouse, the APLP2−/− mouse was crossbred with the SOD1-G37R mouse to generate three distinct mouse lines (SOD1-G37R:APLP2+/+, SOD1-G37R:APLP2 ± and SOD1-G37R:APLP2−/−) and their survival life expectancy was monitored for each sex (Fig. 3). The most dramatic effect of APLP2 gene deletion was observed in the female SOD1-G37R:APLP2−/− mouse whereby its lifespan was significantly increased by 2–3 weeks compared to the SOD1-G37R:APLP2+/+ and SOD1-G37R:APLP2 ± (*p* = 0.0005 and *p* = 0.003, respectively, Fig. 3B). In contrast, life expectancy was unchanged when comparing across the male mouse groups (Fig. 3C). Next, we compared the male to female survival plots between the three different mouse lines and whilst there was no sex-dependent difference in the lifespan between male and female for the SOD1-G37R:APLP2+/+ mouse line (Fig. 3D), the partial deletion of APLP2 gene in the SOD1-G37R:APLP2 ± mouse caused a longer lifespan in the male compared to the female mouse, an effect that was more pronounced in weeks prior to the End-stage of disease progression (*p* = 0.0354, Fig. 3E). In contrast, the mice with homozygous deletion of APLP2 (SOD1-G37R/APLP2−/−) resulted in a significantly extended lifespan by ~ 1.5 weeks in female compared to male mice (*p* = 0.0058, Fig. 3F). Collectively, these results demonstrate how the SOD1-G37R mouse lifespan and associated End-stage progression of this disease, is directly modulated by the extent of APLP2 gene expression and the sex of the mouse.

### Motor performance is dysfunctional in female but not in male SOD1-G37R mouse lacking APLP2 gene expression

**Fig. 4:**
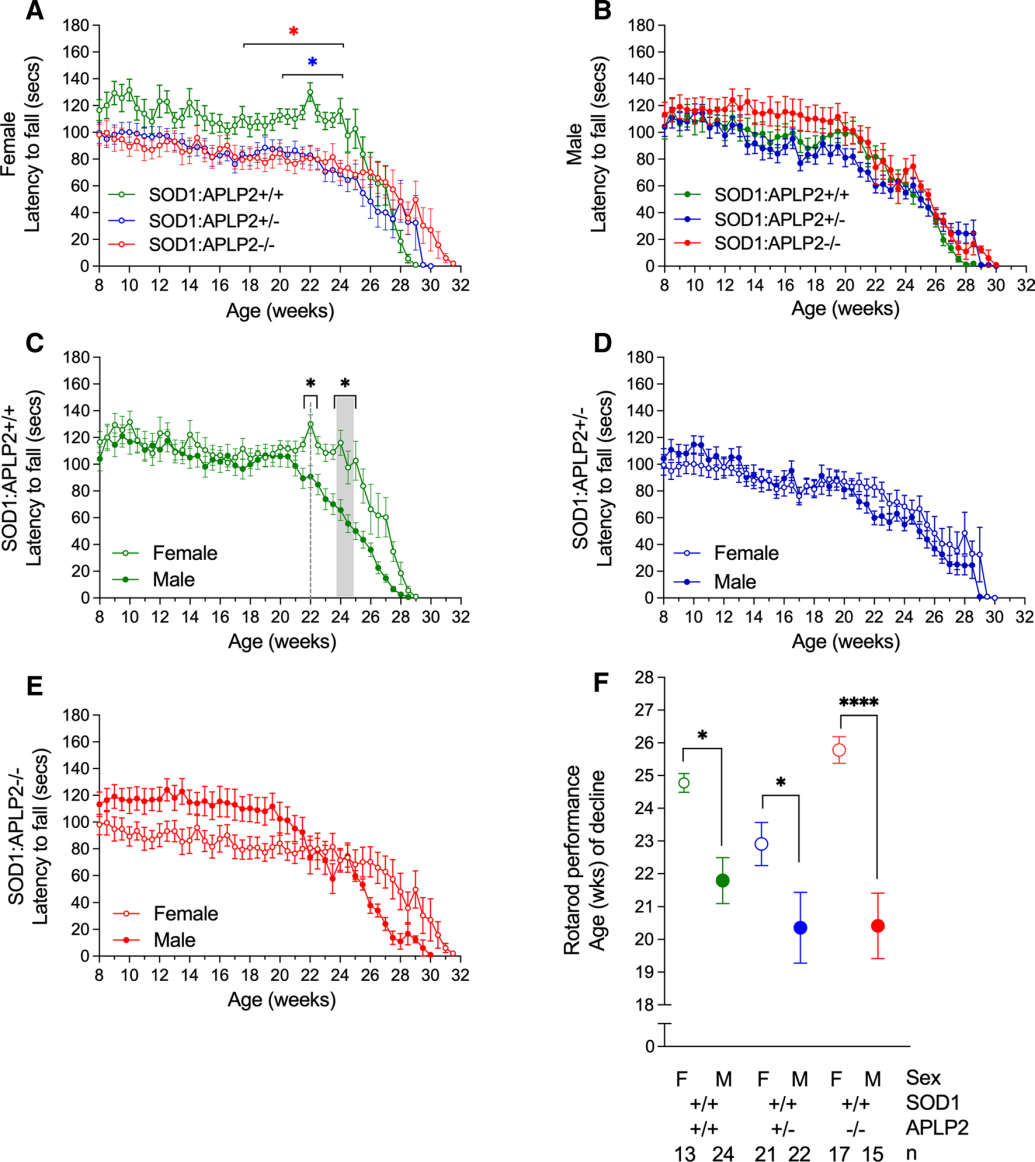
Motor performance dysfunction is delayed in female SOD1-G37R mice with APLP2 gene deletion. Motor performance function was assessed using the rotarod assay and time recorded for latency to fall in both **A** female and **B** male sexes of the SOD1-G37R:APLP2+/+ (green), SOD1-G37R:APLP2 ± (blue) and SOD1-G37R:APLP2−/− (red) mice genotypes. Direct female to male sex comparison was plotted for **C** SOD1-G37R:APLP2+/+, **D** SOD1-G37R:APLP2 ± and **E** SOD1-G37R:APLP2−/− mice cohorts. **F** The age of decline in weeks (wks) for rotarod performance was calculated using the split line regression model in GenStat analysis software. Data are shown as mean ± SEM, analysed using two-way ANOVA with Tukey’s post hoc test for comparison of rotarod performance across the weeks and by student *t* test for female (F) and male (M) sex comparison using the split line regression model, *p < 0.05, **p < 0.01, ****p < 0.000, *n* = 13–24 per group

It is well established that motor performance gradually deteriorates over time in the SOD1-G37R mouse as the disease phenotype progresses over time [42]. Therefore, having established how APLP2 gene deletion significantly prolongs the lifespan of the female SOD1-G37R:APLP2−/− mouse, we next examined whether modulation of APLP2 gene expression affected their motor performance by testing them on an accelerating rotarod instrument [42]. Whilst the female SOD1-G37R:APLP2−/− and SOD1-G37R:APLP2 ± mice performed generally worse off on the rotarod test compared to the SOD1-G37R:APLP2+/+ mouse during the period of 8–25 weeks of age and a statistical difference was found only at 22 and 24 weeks of age (*p* < 0.01, Fig. 4A). In contrast, APLP2 gene deletion in the male SOD1-G37R mouse did not affect their motor performance based on their rotarod testing results (Fig. 4B). When comparing female to male across the three genotype lines, the SOD1-G37R:APLP2+/+ female mouse displayed a general deficit and delay in motor performance compared to males from 21 weeks to end-stage of disease but this was significantly different only at the 22, 24 and 25 week time points (*p* < 0.05, Fig. 4C–E).

To calculate the age of decline in motor performance between the three genotype lines, we opted for a more sophisticated analysis using the GenStat Statistical analysis software package. A split line regression model was used to divide the data into two regression lines to represent two different phases of motor performance. The first phase represents when the mouse performed at equilibrium or there was an incline in the motor performance test and the second phase is depicted when the animal showed a decline in their performance. The model then detects the precise regression breaks between the two regression lines and estimates the age of decline in the motor performance for each animal. Using this analysis strategy, the motor performance in the female mouse was significantly delayed compared to the male mouse in all three genotype lines ranging from ~ 3 weeks in both the SOD1-G37R:APLP2+/+ and SOD1-G37R:APLP2 ± (*p* < 0.05) and by ~ 6 weeks (*p* < 0.0001) in the SOD1-G37R:APLP2−/− groups (Fig. 4F). Given that no differences in the survival and motor performance were observed in the SOD1-G37R:APLP2 groups, all male comparison data for the subsequent behavioural tests will not be discussed and data are provided in the supplementary information.

### Neurological function score is improved in the female SOD1-G37R mouse lacking APLP2 gene expression

**Fig. 5:**
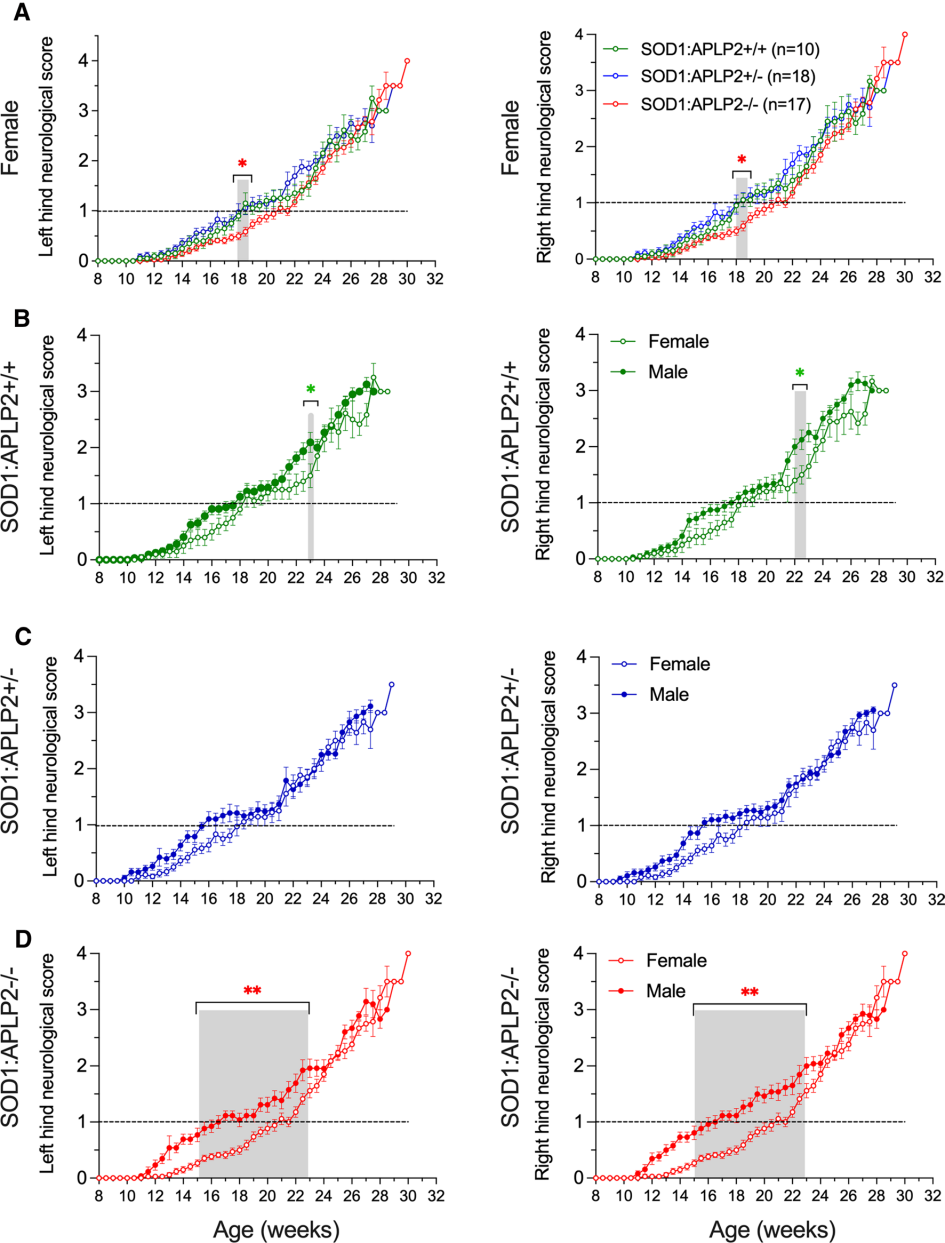
Female SOD1-G37R with APLP2 gene deletion showed delayed neurological function score compared to male mice. The effect of APLP2 gene deletion on the neurological function scores of the SOD1-G37R mouse (SOD1) from 8 weeks of age until disease End-stage for the left and right hindlimbs was assessed for **A** female SOD1-G37R:APLP2+/+ (green), SOD1-G37R:APLP2 ± (blue) and SOD1-G37R:APLP2−/− (red) mice. Left and right hindlimb neurological function scores were compared between the female and male cohorts for **B** SOD1-G37R:APLP2+/+, **C** SOD1-G37R:APLP2 ± and **D** SOD1-G37R:APLP2−/−. Dashed horizontal lines indicate when mice reached a neurological function score of 1, an indication of when disease symptoms are prominent. Values presented as mean ± SEM. Statistical testing using two-way ANOVA with Tukey’s post hoc test, *p < 0.05, **p < 0.01. *n* = 10–19

The effect of APLP2 deficiency on disease severity was examined by calculating the neurological function score based on the ALS TDI criteria [44, 49]. Mice at the early disease stage (~ 8 weeks) were suspended by their tails and their left and right hindlimb extensions were monitored and scored up until End-stage disease. As expected, as disease symptoms develop and progress in this MND mouse model, left and right hindlimb extensions were impaired in all three SOD1-G37R:APLP2 genotypes and this was indicated by the progressive increase in the neurological scores for both female and male groups (Fig. 5A and S2). We next compared the neurological scores for age and observed decreases in the neurological scores in the female SOD1-G37R:APLP2−/− mouse between 17 and 20 weeks of age, and significant at 18 weeks of age when compared with both SOD1-G37R:APLP2+/+ and SOD1-G37R:APLP2 ± for both left and right hindlimbs (Fig. 5A). In comparison, the male SOD1-G37R:APLP2−/− mouse exhibited significantly improved neurological scores (*p* < 0.05) in the right hindlimb at 24 and 25 weeks of age (Figure S2).

Consistent with the rotarod test, a sex comparison of the SOD1G-37R:APLP2+/+ (Fig. 5B) showed a significantly better neurological function score in females at 23 weeks for left hindlimb and at 22 weeks for right hindlimb. The delay in symptoms onset was more pronounced for female SOD1G-37R:APLP2−/−, which was extended by ~ 4 weeks to reach a neurological score of 1 (*p* < 0.01, Fig. 5C). No differences were observed for the heterozygous SOD1G-37R:APLP2 ± group when sexes were analysed separately for either left or right hindlimbs (Fig. 5D). In summary, APLP2 deletion in the SOD1G-37R mouse is associated with altered neurological function since symptom onset is delayed and disease progression is altered, effects that were more pronounced in female mice.

### Motor performance is improved prior to disease symptom onset in the female but not in male SOD1-G37R mouse lacking APLP2 gene expression

**Fig. 6:**
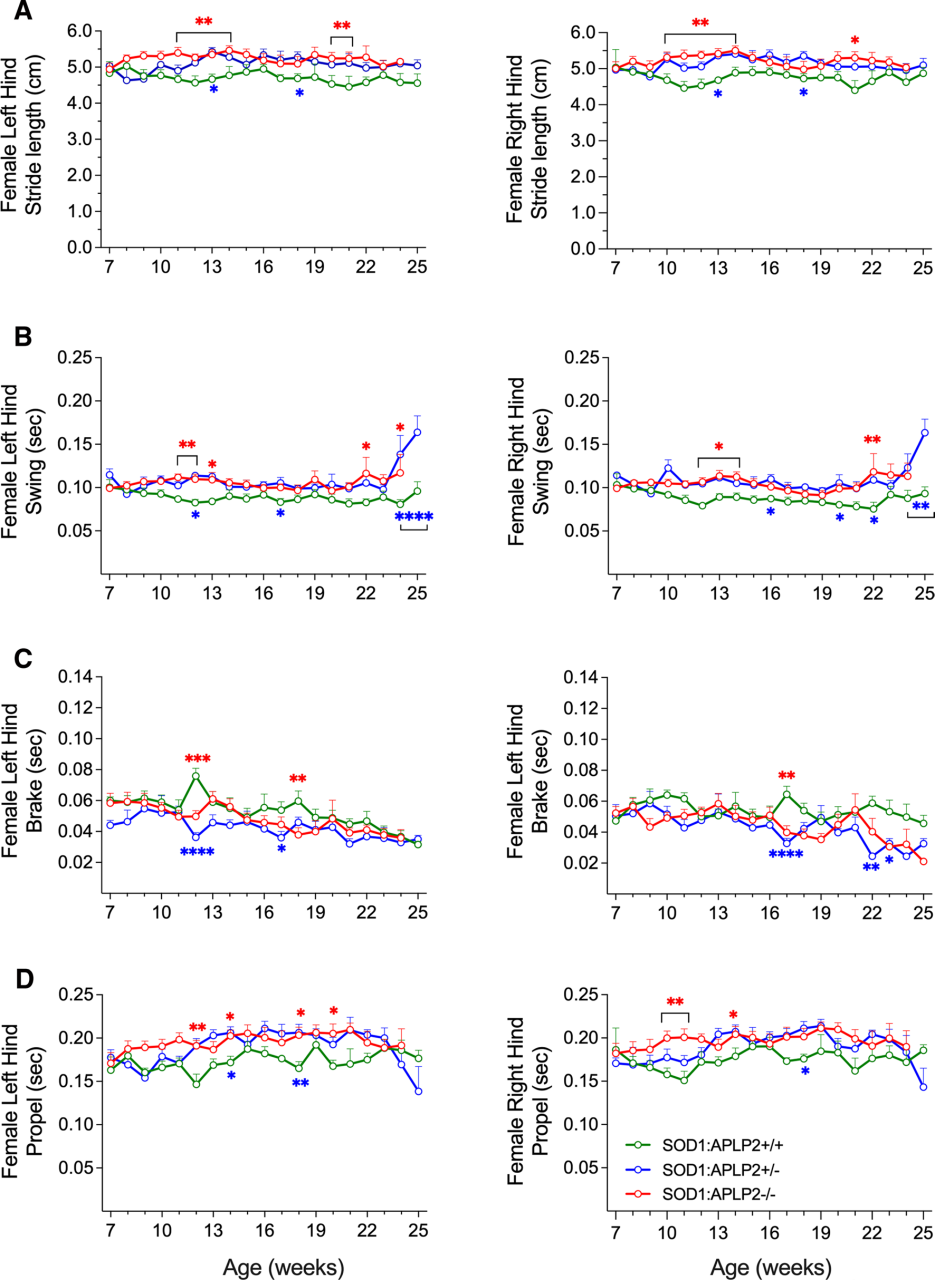
APLP2 deletion improves various hindlimb gait indices of female SOD1-G37R mice prior to symptom onset. DigiGait analysis of left and right hindlimbs recorded at weekly intervals from 7 to 25 weeks of age and measurements for **A** stride length, **B** swing, **C** brake and **D** propel for female mice of SOD1:APLP2+/+ (green), SOD1:APLP2 ± (blue) and SOD1:APLP2−/− (red) genotypes. Data presented as mean ± SEM. Statistical testing comparing to SOD1-G37R:APLP2+/+ using two-way ANOVA with Tukey’s post hoc test, *p < 0.05, **p < 0.01, ****p < 0.0001. *n* = 5–8

Disease progression in MND can be characterised by gait deficits. Therefore, we used the DigiGait platform to monitor the mice as it provides a very comprehensive analysis of 54 different gait parameters. Whilst three different speeds (10 cm/s, 15 cm/s and 20 cm/s) were initially tested, only the data collected from the 15 cm/s speed are presented since this speed allowed the mice to perform successfully on the instrument from early onset and until the late phase of disease detection. However, as the SOD1-G37R:APLP2 mouse lines progressed through to disease End-stage (data not shown), they were less likely to successfully perform on the DigiGait platform especially the male SOD1-G37R:APLP2−/− mouse which were unable to complete the assessment task beyond 23 weeks of age. Of the 54 parameters that were assessed by DigiGait, significant changes were identified in 35 parameters and at different ages throughout the disease assessment period. We observed similar gait and body movement patterns between the different male SOD1-G37R:APLP2 genotypes for all limbs (Figure S3). We also detected similar gait patterns for the left and right fore-limbs of the female mice cohort. However, a significant difference was observed in the left and right hindlimbs of the female groups for stride length, swing, brake and propel. Therefore, only the hindlimb data of the female groups are shown. The female SOD1-G37R:APLP2−/− mouse displayed a significant improvement in stride length for both the left and right hindlimbs beginning at 11 weeks of age, and it remained higher than their female SOD1-G37R:APLP2+/+ counterparts as the disease progressed (Fig. 6). The female SOD1-G37R:APLP2 ± mouse also exhibited a significant improvement in stride length, however, this was only significant at the later 12 and 18 weeks of age. Significant improvement in swing, brake and propel indices were also calculated at 11 and 12 weeks for the SOD1-G37R:APLP2−/− and SOD1-G37R:APLP2 ± female mouse, respectively. Overall, the results from DigiGait data are in agreement with the neurological scores, indicating a lack or reduction in APLP2 expression is associated with improved gait function in the female SOD1-G37R mouse but they do not align with the rotarod data which indicated that the APLP2 KO female mouse had a worse motor phenotype at the earlier ages.

### No improvement in motor neuron loss, astrogliosis or microgliosis in the lumbar spinal cord in SOD1-G37R mouse lacking APLP2 expression

**Fig. 7:**
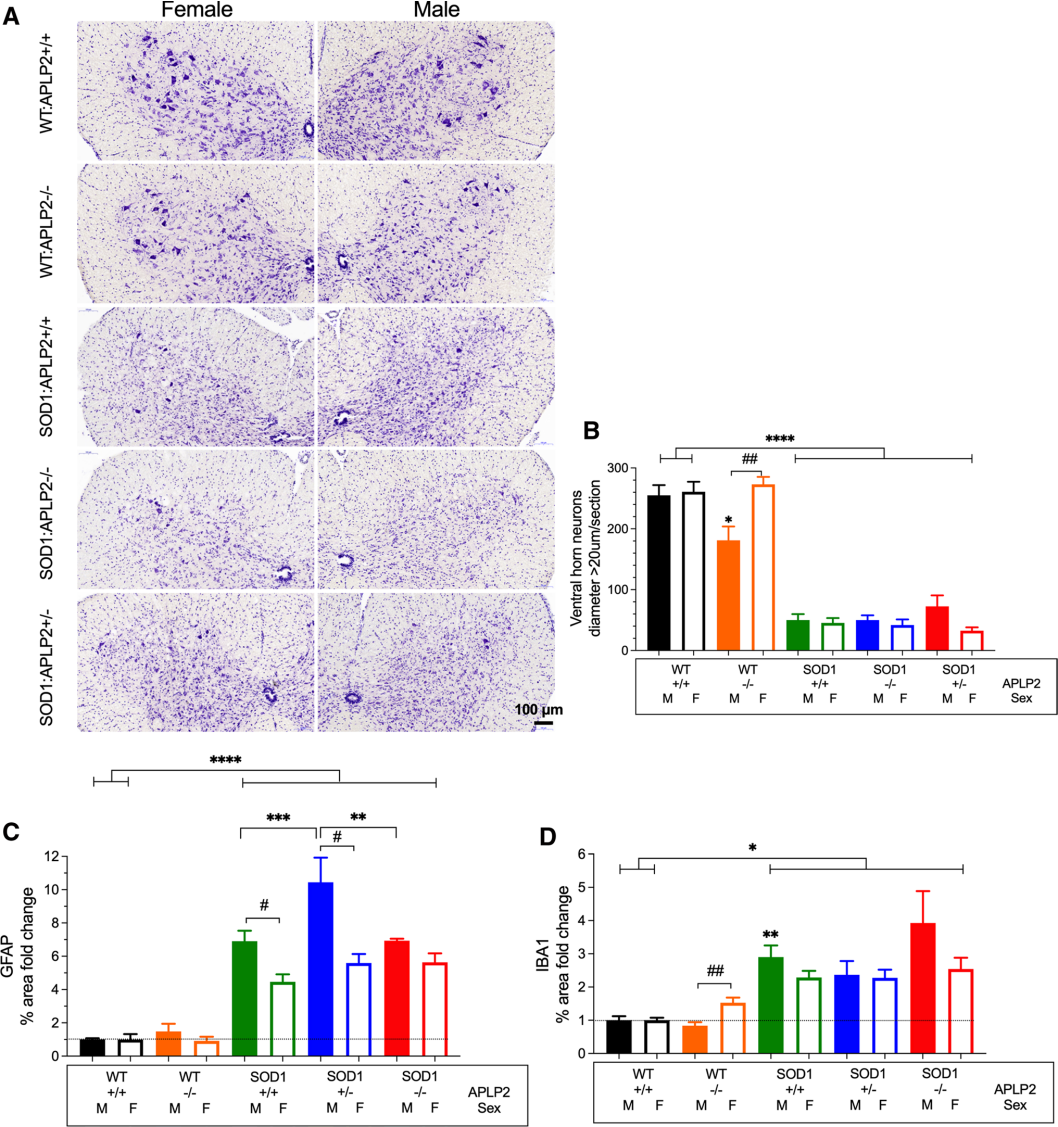
APLP2 gene deletion in SOD1-G37R mice does not affect motor neuron survival, astrogliosis and microgliosis. **A** Representative micrographs of motor neurons in the ventral horn of the spinal cord tissue sections stained with cresyl violet of female and male mice cohorts for WT:APLP2+/+, WT:APLP2−/−, and disease End-stage for SOD1-G37R:APLP2+/+, SOD1-G37R:APLP2 ± and SOD1-G37R:APLP2−/− mice. Scale bar = 100 μm. **B** The number of spinal neurons counted in seven sections with a diameter > 20 µm and located in the ventral horn region and situated between L4 and L5 spinal cord segments were plotted for male (M) and female (F) mice. Quantitative analysis of the ventral horn lumbar spinal cord tissue sections immunolabelled with **C** anti-GFAP and **D** anti-IBA1 antibodies of WT:APLP2+/+, WT:APLP2−/−, SOD1-G37R:APLP2+/+, SOD1-G37R:APLP2 ± and SOD1-G37R:APLP2−/− mice at disease End-stage. Data show the percentage fold change of areas positively immune-stained with GFAP and IBA1 and then normalised to the respective female and male WT:APLP2+/+ mice groups. Values presented as mean ± SEM. Statistical testing by one-way ANOVA with Bonferroni’s post hoc test were used for genotype comparison to respective female and male WT:APLP2+/+ mice groups, *p < 0.05, **p < 0.01, ***p < 0.001 and ****p < 0.0001, and a student *t* test was used for comparison between sexes, ^#^*p* < 0.05, ^##^*p* < 0.01. *n* = 5–6

We next evaluated whether the delayed motor performance measured in the female SOD1-G37R:APLP2−/− mouse may be explained by motor neuron degeneration in the spinal cord. To address this, α-motor neurons, which are the most vulnerable motor neuron in MND were identified in spinal cord sections by Nissl staining and their size and quanta were determined (Fig. 7A). Since the diameter size of the larger α-motor neurons in transgenic SOD1 mouse strains range from 18 to 37 μm [50], we classified neurons with a diameter size greater than 20 µm as α-motor neurons for quantitative analysis. As expected, the transgenic SOD1-G37R mouse contained four- to five fold lower numbers of α-motor neurons and this was significantly different to their WT littermate controls (Fig. 7B). There was no difference in the number of α-motor neurons in the SOD1-G37R:APLP2−/− and SOD1-G37R:APLP2 ± mice when compared to SOD1-G37R:APLP2+/+ for both female and male groups. Interestingly, APLP2 gene deletion caused a 30% reduction in α-motor neurons numbers in the male mouse and this was significantly different compared to both its WT litter mate control and to the female WT:APLP2−/− mouse, but this sex difference was not recapitulated in the SOD1-G37R mouse model (Fig. 7B). Taken together, these results suggest that APLP2 deficiency in the SOD1-G37R mouse does not affect motor neuron viability in this MND model.

Astrogliosis is a well-characterised pathological feature of MND and has been reported in human ALS patients and transgenic animal models of familial ALS [51, 52]. Therefore, we examined if APLP2 expression modulated the activation state of astrocytes by counting and quantitating the percentage area of GFAP positive immunoreactive signals in the spinal cord tissue sections across the different mouse lines (Figure S4A). Whilst the percentage change in the GFAP-stained area was significantly higher in both the female and male groups for SOD1-G37R:APLP2+/+, SOD1-G37R:APLP2−/− and SOD1-G37R:APLP2 ± mice when compared with their respective WT:APLP2 littermate controls, a sex-dependent comparison of GFAP expression in the different genotypes revealed a significant reduction in the female compared to male groups of both SOD1-G37R:APLP2+/+ and SOD1-G37R:APLP2 ± but not SOD1-G37R:APLP2−/− (Fig. 7C).

Microglial activation, which parallels MND progression [51], was examined in the ventral horn of the spinal cord tissue sections by immunohistochemistry analysis (Figure S4B). Microglial activation, which was assessed by calculating the percentage area change of Iba1 immunoreactivity, was significantly increased in the SOD1-G37R:APLP2 female and male cohorts compared to the WT littermate controls (Fig. 7D). The only sex differences observed were in the WT:APLP2−/− mouse where by the females displayed significantly higher levels of microglial activation compared to male mice (Fig. 7D).

### APP protein level is reduced in the SOD1-G37R mouse lacking APLP2 expression

**Fig. 8:**
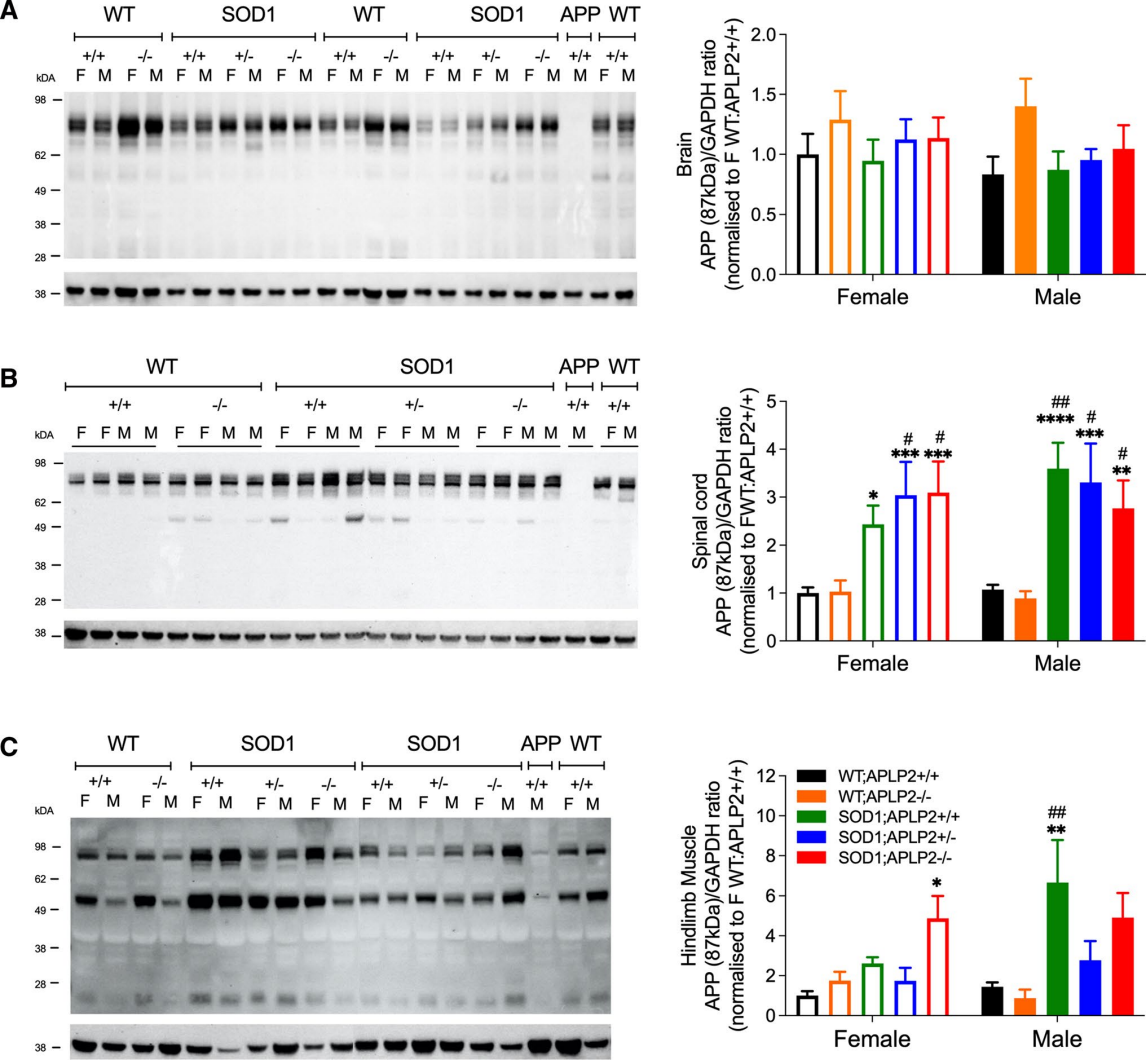
Genetic deletion of APLP2 gene decreases APP levels in the spinal cord of SOD1-G37R mice. Representative immunoblots and densitometric quantification of the full length ~ 87 kDa APP band in the **A** brain **B** spinal cord and **C** hindlimb muscles of SOD1-G37R:APLP2+/+, SOD1-G37R:APLP2 ± , SOD1-G37R:APLP2−/− mice at disease End-stage and WT:APLP2+/+, WT:APLP2−/− age-matched littermate controls. APP−/− and WT:APLP2+/+ mouse tissue samples were used as negative and positive controls, respectively. Values presented as mean ± SEM. Statistical testing by one-way ANOVA with Bonferroni’s post hoc test comparison, *p < 0.05, **p < 0.01, ***p < 0.001 and ****p < 0.0001 compared to WT:APLP2+/+ and ^#^*p* < 0.05 and ^##^*p* < 0.01 compared to WT:APLP2−/− mice. *n* = 4–6

APP protein has been shown to have several important physiological roles [53, 54] and its expression is altered in different pathological diseases including MND [22] suggesting that it may have important roles in modulating disease progression. Since APLP2 and APP belong to the same gene family, we investigated if APLP2 deficiency in the SOD1-G37R mouse affected the APP protein expression level or patterns in various organs affected by MND. Brain, spinal cord and hindlimb muscles were harvested at End-stage of disease from SOD1-G37R:APLP2 and age-matched WT:APLP2 littermates and endogenous APP protein expression quantitated by Western blotting analysis. APP protein expression level in whole brain lysate samples was similar across all mouse genotypes and between female and male cohorts too (Fig. 8A).

In contrast, the spinal cord lysates contained significantly higher APP protein expression levels (~ 2–3-fold) in all SOD1-G37R:APLP2 genotypes for both female and male groups compared to the WT genotypes, respectively (Fig. 8B). To support this Western blot data analysis, immunohistochemistry was performed and the intensity of APP immunoreactivity in the ventral horn region of the lumbar spinal cord sections was quantitated and expressed relative to the WT control group (Figure S5A and S5B). In agreement with the Western blot analysis, we observed a significant increase in total APP immunoreactivity relative to the WT control groups, in both sexes of the SOD1-G37R:APLP2+/+ mouse (Figure S5B). Whilst the level of APP immunoreactivity remained significantly elevated in the male SOD1-G37R:APLP2 ± and SOD1-G37R:APLP2−/− mouse compared to the WT control group, the APP levels were partially decreased and significantly different to the SOD1-G37R:APLP2+/+ group (Figure S5B). In contrast to the male mice, the female mice displayed and unexpected finding where by mice lacking one or both APLP2 alleles displayed lower levels of APP immunoreactivity compared to both the female SOD1-G37R:APLP2+/+ mouse and male SOD1-G37R:APLP2−/− and SOD1-G37R:APLP2 ± , respectively, and levels were not different to WT groups too (Figure S5B). These contrasting results may be explained by the differences in sample preparation since the whole spinal cord from the lumbar region was used for Western blotting and only the small localised ventral horn region, which accounts for ~ 20% of the total surface area within the spinal cord tissue, was only assessed for immunohistochemistry analysis suggesting that focal APP expression levels differs within localised regions of the spinal cord in these MND mouse tissue.

In contrast to the neural tissue where an 87 kDa band was the major APP species present, the tissue lysates prepared from hindlimb muscle contained 2 prominent APP species with molecular weights of 87 and 55 and a third band that was both fainter and smaller at 25 kDa band (Fig. 8C). The band intensity of the largest 87 kDa APP species was significantly higher in the female SOD1-G37R:APLP2−/− (~ 5.3-fold) and male SOD1-G37R:APLP2+/+ (~ 4.8-fold) mice when compared to WT:APLP2+/+ mice (Fig. 8C). Further quantitative analysis of the smaller 55 kDa and 25 kDa APP immunoreactive bands were found to be not statistically different (Figure S5C and S5D).

### APLP2 gene deletion exacerbates NMJ denervation in male SOD1 mice and improves NMJ innervation in the female SOD1-G37R mouse

**Fig. 9:**
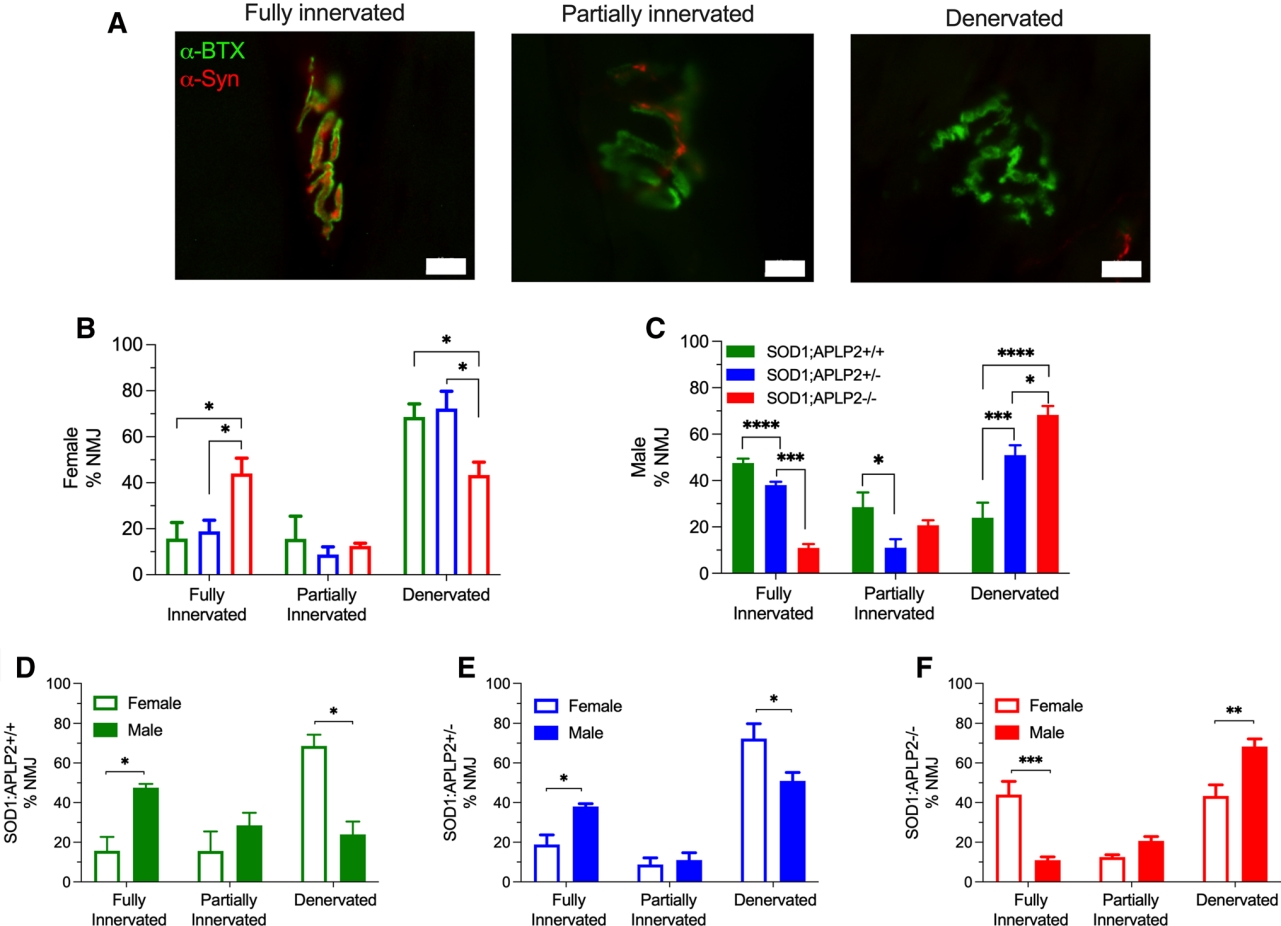
APLP2 gene deletion ameliorates NMJ denervation in female SOD1 mice. **A** Representative images of colocalization between pre-synaptic and post-synaptic marker defined as fully innervated, partially innerved and denervated NMJ. Scale bar = 10 μm. Arrows indicated collapsed and fragmented NMJ. Calculated percentages of fully innervated NMJ (> 20% colocalisation), partially innervated NMJ (10–20% colocalisation) and denervated NMJ (< 10% colocalisation) in three muscle Sects. (100 μm apart) for **B** male and **C** female SOD1-G37R:APLP2+/+, SOD1-G37R:APLP2−/− and SOD1-G37R:APLP2 ± mice groups. NMJ innervation status was also compared between sexes for **D** SOD1-G37R:APLP2+/+, **E** SOD1-G37R:APLP2 ± and **F** SOD1-G37R:APLP2−/−. Data presented as mean ± SEM and statistical testing by one-way ANOVA with Bonferroni’s post hoc test, *p < 0.05, **p < 0.01, ***p < 0.001, ****p < 0.0001. *n* = 3–4

Both APP and APLP2 are physiologically expressed in motor neurons and muscle tissue and especially in the NMJ where they provide important roles in its formation and maturation [30, 55–57]. As expected, NMJ in the extensor digitorum longus of SOD1-G93A mouse, an alternative MND mouse model, displayed significant denervation whilst ablation of the APP gene in this mouse model reversed this phenotype and a significant decrease in the proportion of denervated NMJs was observed [22]. In our studies, we examined the GA muscle at End-stage of disease and the NMJ was identified by indirect immunofluorescence and quantitating the extent of colocalization between alpha-bungarotoxin and synaptophysin (Fig. 9A). Based on their innervation of the motor end plate, NMJs were classified into three groups, fully innervated (> 20% colocalisation), partially innervated (10–20% colocalisation) or denervated (< 10% colocalisation), where denervation of NMJs is revealed by a reduction in the number of ‘pretzel-shaped’ NMJs and these are accompanied by the collapse and fragmentation of NMJ structures (Fig. 9A). Our results revealed that the female SOD1-G37R:APLP2−/− mice displayed a significantly higher proportion of fully innervated NMJs and a significantly decreased proportion of denervated NMJ when compared to both the SOD1-G37R:APLP2+/+ and SOD1-G37R:APLP2 ± mice groups (Fig. 9B). In contrast, a mirror opposite effect was observed for the male SOD1-G37R:APLP2−/− mouse whereby these mice had significantly lower proportion of fully innervated NMJs and a significantly higher proportion of denervated NMJs compared to both SOD1-G37R:APLP2+/+ and SOD1-G37R:APLP2 ± mice groups (Fig. 9C). Upon further analysis examining these sex-based differences on the NMJ innervation pattern, we determined that there was a threefold significant lower proportion of the fully innervated NMJs with a concomitant threefold significant higher proportion of denervated NMJs in female compared to male SOD1-G37R:APLP2+/+ mice (Fig. 9D). In the SOD1-G37R:APLP2 ± mouse, which partially expresses the APLP2 gene, the NMJ innervation pattern closely mirrored the significant changes observed when comparing the female and male SOD1-G37R:APLP2+/+ mice (Fig. 9E). In contrast, a significantly higher proportion of fully innervated NMJs and a significantly lower proportion of denervated NMJs were calculated in the female SOD1-G37R:APLP2−/− mouse compared to its male group (Fig. 9F). Taken together, our findings reveal a potential redundant role of APLP2 at the NMJ innervation and its component involvement may explain the delayed disease onset observed in the female SOD1-G37R:APLP2−/− mouse.

### APLP2 gene deletion exacerbates muscle atrophy in male SOD1 mice but ameliorates atrophy in the female SOD1-G37R mouse

**Fig. 10:**
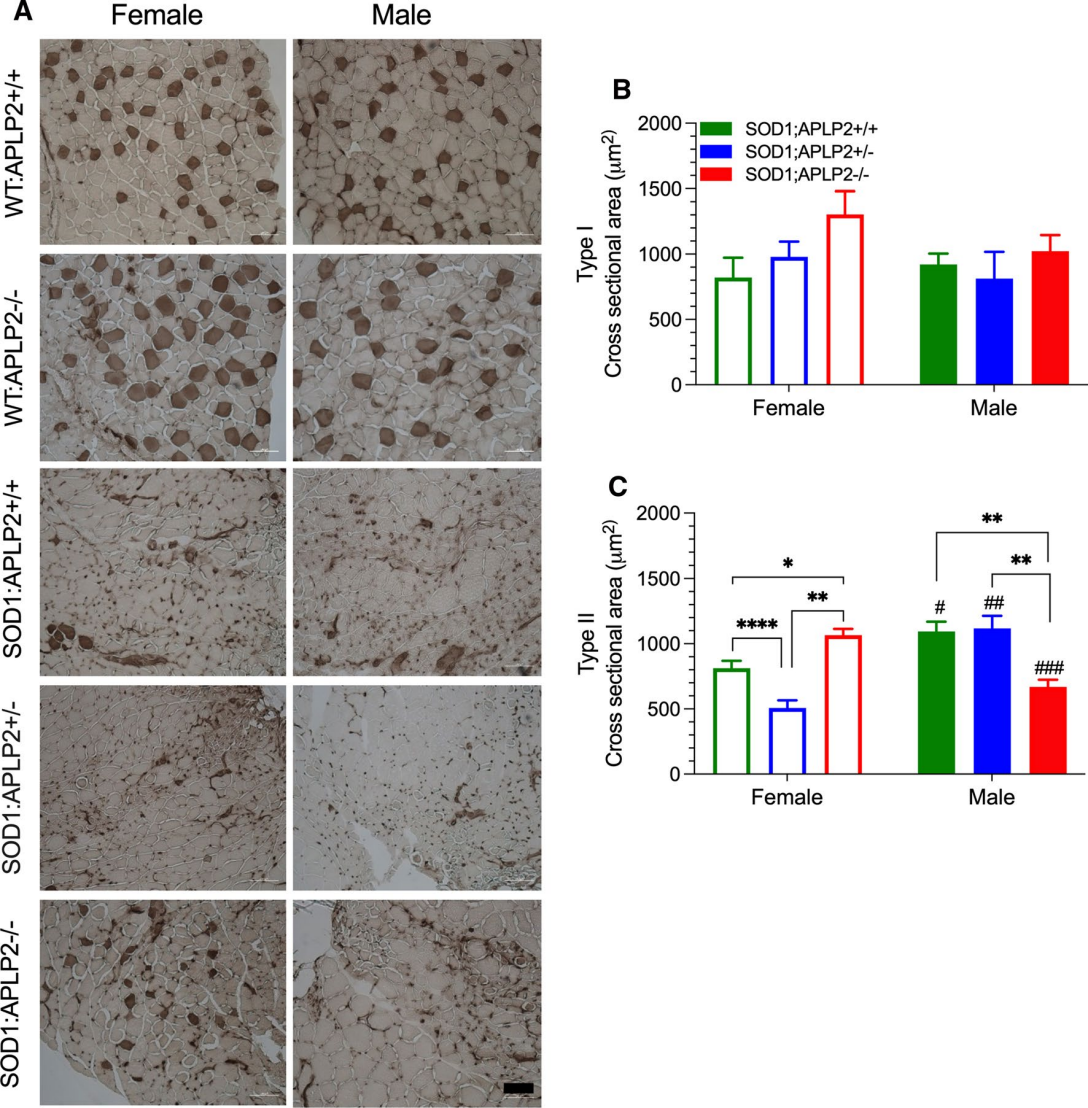
Genetic deletion of APLP2 expression in female mutant SOD1-G37R mice improves muscle fibre atrophy. **A** Representative micrographs of gastrocnemius muscle cross-sections stain with ATPase (pH 4.3) from female and male SOD1:APLP2+/+, SOD1-G37R:APLP2 ± , SOD1-G37R:APLP2−/− at the End-stage of disease and their age-matched WT littermates control WT:APLP2+/+ and WT:APLP2−/−. Quantification of **B** slow twitch/type I and **C** fast twitch/type II myofibres cross-sectional area for both female and male groups of SOD1:APLP2+/+, SOD1-G37R:APLP2 ± , SOD1-G37R:APLP2−/− at disease End-stage. Data present as mean ± SEM and statistical testing by one-way ANOVA with Bonferroni’s post hoc test were used for genotype comparison within the same sex, *p < 0.05, **p < 0.01, ***p < 0.001, ****p < 0.0001, and student t test was used for sex comparison within the same genotype, ^#^*p* < 0.05, ^##^*p* < 0.01, ^###^*p* < 0.001. *n* = 4–6. Scale bar = 100 μm

To investigate whether the deteriorating motor function in SOD1-G37R:APLP2 mice coincides with the progressive and widespread wasting observed in the hindlimb muscles, we performed transverse sections of the gastrocnemius muscle and the muscle myofibre types identified by ATPase staining and their cross-sectional area measured and binned into separate slow twitch Type 1 and fast twitch Type II fibre types according to their size (Fig. 10). The muscle cell morphology in all different SOD1-G37R:APLP2 mouse lines at End-stage of disease progression were significantly compromised since the large muscle myofiber cell structures were dramatically smaller in appearance compared to the WT:APLP2+/+ and WT:APLP2−/− muscle tissue (Fig. 10A). Quantification of the cross-sectional area (CSA) of these myofiber cells between the SOD1-G37R:APLP2 mouse genotypes showed no change in the presence of the type I slow twitch muscle fibres and no difference was observed between female and male groups when comparing across the three SOD1-G37R:APLP2 genotypes examined (Fig. 10B). In contrast, CSA type II muscle fibres, the most vulnerable type of muscle fibres in MND pathogenesis [58–60], were significantly reduced by ~ 40% in the male SOD1-G37R:APLP2−/− mouse compared to both SOD1-G37R:APLP2+/+ and SOD1-G37R:APLP2 ± mice groups (Fig. 10C). The CSA Type II fibres were significantly increased in the female SOD1-G37R:APLP2−/− mouse compared to SOD1-G37R:APLP2+/+ (~ 24%) and SOD1-G37R:APLP2 ± mice (~ 53%, Fig. 10C). Whilst no changes were observed in the CSA of Type II fibres for male SOD1-G37R:APLP2 ± mice, a significant reduction in CSA was observed for female SOD1-G37R:APLP2 ± (~ 42%) when compared with SOD1-G37R:APLP2+/+ mice. Sex comparison of CSA across the different genotypes revealed a significant reduction in CSA in female groups of SOD1-G37R:APLP2+/+ and SOD1-G37R:APLP2 ± for Type II myofibres, but a significant reduction was observed in the male groups of SOD1-G37R:APLP2−/− mice (Fig. 10C). These results parallel the NMJ results and highlight the potential involvement of skeletal muscle deficits of APLP2 deficiency affecting lower motor neuron pathology in SOD1 mice.

## Discussion

The triggering event that causes MND is still largely unknown and hotly debated but there is good evidence that APP gene family play some important role in the pathology of this disease [22, 27, 61]. This notion is strongly supported by our new findings showing APP and APLP2 protein expression are significantly elevated in a sex-dependent manner in the spinal cord tissue isolated from post-mortem human MND cases compared to normal age-matched control subjects, with an interestingly higher levels of APLP2 in female MND patients compared with male patients. This sex difference could help in understanding the protective factors conferred by female sex and could potentially aid in developing therapies. To further explore the role of these genes in MND, the SOD1-G37R mouse model was utilised since it replicates many of the human phenotypes including premature death due to the significant damages associated with both the neuron and muscle cell physiology [42, 43, 62]. Like the human MND patients, we also identified a sex-dependent increase in both APLP2 and APP protein expression levels in the spinal cord samples of SOD1-G37R mouse and only at End-stage disease of this debilitating MND model compared to the control group. But unlike the human MND data, APLP2 protein levels was elevated to a similar extent between female and male groups whilst APP was more significantly elevated in the male compared to the female mouse group. The difference between human and mouse MND models may be explained in part by the greater increase in astrogliosis in the male compared to female SOD1-G37R mice groups (Fig. 7C) since previous studies have demonstrated astrocytes to be sexually dimorphic in response to an inflammatory challenge [63], oxygen glucose deprivation [64] and expression of enzymes involved in steroid synthesis and metabolism [65] thus contributing to the existence of sex differences in pathological diseases including MND.

Apart from MND, the upregulation of APP expression, which has also been shown in brain neurons following TBI insults in both human and animal induced models [66–68] corresponded to a neuroprotective response in the localised injured region [23, 68]. A follow-up study demonstrated the therapeutic potential of APP by administering the smaller sAPPα by intracerebroventricular injection into TBI injured rats resulting in decreased axonal injury, reduced apoptosis and improved functional outcomes [69]. Additional evidence in support of a neuroprotective role of APP following injury was demonstrated by the increased susceptibility of to TBI injury in mice lacking APP gene which also developed poorer motor and cognitive outcomes [70], whilst administration of sAPPα or APP96-110 in these mice successfully prevented the TBI induced pathologies [71, 72]. Based on these TBI injury models, it was proposed that the APP protein expression upregulation in the nerve terminals following brain injury has an important function in adaptive and neuroprotective responses [23, 71]. Unlike TBI, it does not appear that APP has a neuroprotective role in MND since genetic ablation of APP in the SOD1-G37R mouse was associated with a decrease in the larger motor neuron death and an ameliorated NMJ denervation [22]. The neuroprotective role of APP may be tissue site specific since MND disease is mostly localised to the spinal cord whilst the brain is the major site associated with TBI injuries. Our study supports this notion since increased APP expression, was only seen in the SOD1-G37R mouse spinal cord at the End-stage of disease, the major site of motor neuron degeneration in MND, accompanied with prominent astrogliosis and microgliosis. Nevertheless, our new findings further illustrate the broad functional physiological roles of APP in both health and especially in disease environments with its multiple and distinct actions being dependent on the type, duration, timing and especially the tissue site of disease or injury.

Studies examining the effect of genetic ablation of APLP2 in the mouse model has led to the successful discoveries of a redundant role of APLP2 in a number of clinical diseases such as myopia [39], retinal synaptopathy, a congenital stationary night blindness condition [41] and in corneal epithelial wound healing [73]. It is not surprising that APLP2, which is a part of the APP gene family, also displays trophic properties. For example, exogenous treatment with recombinant APLP2 of chick sympathetic neurons promoted neurite outgrowth [31], whilst elevated levels of cellular APLP2 were associated with increased cellular growth of several cancer types including pancreatic cancer [74, 75], colon cancer [76], breast cancer [77] and Ewing’s sarcoma cell lines [78, 79]. Whether APLP2 also has a role in MND was investigated by crossbreeding the APLP2−/− mouse with the SOD1-G37R mouse and this generated viable progeny expressing SOD1-G37R:APLP2−/− and SOD1-G37R:APLP2 ± mouse lines which lacked both or one APLP2 allele, respectively. In addition, a clear strength of our body of work is that both female and male sexes across the different animal lines were investigated whilst most studies typically use only a single sex model. Importantly, our approach resulted in the discovery of how APLP2 gene ablation in the SOD1-G37R mouse resulted in a significant increase in survivability in the female but not male mouse demonstrating a clear sex-based difference in MND. Several sex differences in both behavioural and biochemical changes between the SOD1:APLP2 mouse genotypes were detected, and these are summarised in Table 2. For example, the female SOD1-G37R:APLP2−/− mouse displayed delayed motor dysfunction during disease progression compared to their male counterpart. Perhaps the clearest and most striking aspect of our study is the contrasting result of the SOD1:APLP2 ± mouse as the male group displayed a longer survivability compared to the female group, whilst the opposite result was seen in the SOD1:APLP2−/− mouse. Therefore, it appears the allelic expression of APLP2 may contribute to the compensatory roles of other APP family members. This is supported by previous studies demonstrating how deleting a APP gene family, either independently or in combination, in knockout mouse models revealed non-redundant roles of APP and APLP2 genes, thus demonstrating whole or partial expression of these genes is critical for animal survivability [56, 80]. Therefore, the importance of experimenting in both sexes is clearly evident as demonstrated in this study and contrasts with a previous report that investigated only the female SOD1-G93A mouse cross bred with a genetic ablation of APP and showing no significant extension in survivability but they did display a significant delay in disease onset or progression [22].

**Table 2:**
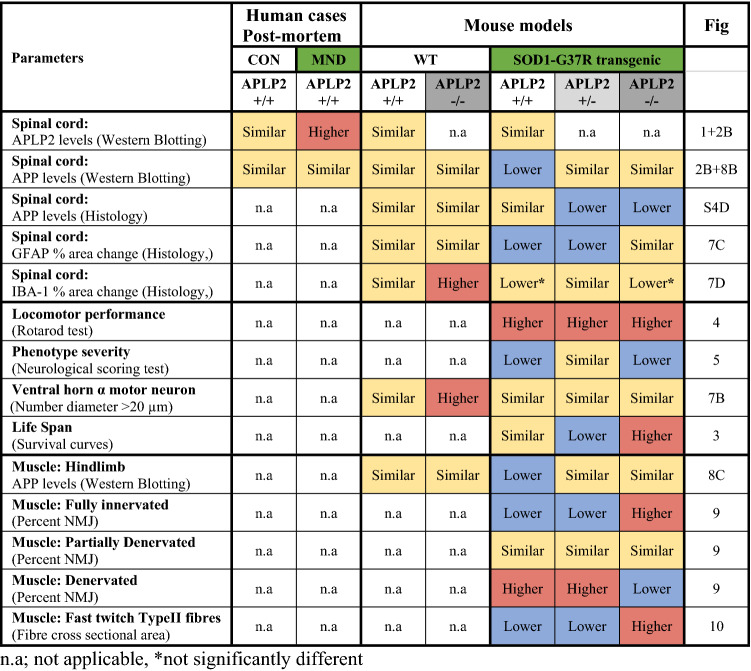
Phenotype and biochemical changes that are significantly different in the female compared to male groups across human post-mortem and mouse models (End-stage) for MND

*n.a* Not applicable

*Not significantly different

The expression of APP protein was reported to be elevated in muscle of human patients with MND and in a the SOD1-G93A mouse model of MND [27] over time as the disease progressed, however, we did not see any detectable changes in either APP and APLP2 protein levels in skeletal muscle of the SOD1-G37R mouse model for MND throughout disease progression. This could be explained by the differences in MND mouse model examined since the pattern of SOD1 protein expression differs between tissue locations with higher concentrations typically detected in the spinal cord compared to hindlimb skeletal muscle (GA) [62, 81], or that the severity of this disease is associated with the level of tissue SOD1 protein expression [43]. Since both APP and APLP2 expressions are important in NMJ formation and maintenance during development [30, 56], their functional roles in MND may be associated with the phenomenon of NMJ degeneration being the triggering event in MND pathogenesis. Upon further examination of the morphology of muscles in the SOD1-G37R mice lacking partial or total APLP2 expression, revealed these mice exerted beneficial effects on skeletal muscle function, which was demonstrated by attenuated NMJ denervation, reduced muscle atrophy, ameliorated motor performance decline and together, this led to extended survival times, an effect that occurred in a sex-dependent manner. The biochemical changes at the NMJs whereby significant improvement and reduction in denervation and muscle atrophy in the female SOD1-G37R:APLP2−/− mouse compared to SOD1-G37R:APLP2 wild-type littermates were observed, suggest APLP2 may be directly involved with NMJ denervation in MND. Whilst the female SOD1:APLP2 ± mouse survival time was shorter compared to their male counterparts, this was associated with a reduction in NMJ innervation and muscle fibre cross-sectional area. Furthermore, in female SOD1:APLP2 ± and SOD1:APLP2−/− mice, levels of APP were lower in spinal cord lysates when compared with their respective male groups although APP levels were significantly upregulated in the SOD1-G37R mouse.

Whilst all female SOD1-G37R:APLP2 genotypes demonstrated a higher locomotor performance, only female SOD1-G37R:APLP2 wild types and SOD1-G37R:APLP2 heterozygous animals showed less severe astrogliosis in the spinal cords, highlighting the disparity between motor neurons physiology and motor functions. Using ChAT antibody as a marker for motor neurons in the ventral horn of the spinal cord, we observed both APP and APLP2 were expressed in motor neurons, with selectivity in the expression of APP only in certain neurons (Figure S6). These results demonstrate that whilst APP and APLP2 share functional similarities at the NMJ in MND pathogenesis, their expression may also have distinctly different effects in the CNS and PNS. Therefore, to determine whether these protective effects are due to ablation of APLP2, future studies utilising a viral mediated expression of APLP2 in motor neurons and muscle cells would help to further underpin this idea and help to further clarify the underlying mechanisms of APLP2, and/or APP for that matter, in muscle vulnerability during development of the disease process. Nevertheless, our new findings support the model that APLP2 may be associated with NMJ denervation in MND and that the distinct functional differences observed for APP and APLP2 suggests they may affect alternate signalling pathways in the pathophysiology of MND. Moreover, these sex-based differences are not surprising since we previously reported how the female APLP2−/− mouse had better motor functions compared to its male counterpart and to its WT littermates during ageing, with a higher number of α-motor neuron populations measured (Fig. 7C) [82]. However, the major sex differences we observed were limited to the End-stage of disease progression as little or no effects of APLP2 gene ablation were seen at the earlier symptomatic and pre-symptomatic stages in our MND mouse model classification which suggests little involvement of APLP2 prior to severe disease onset. Based on these observations, future studies examining the gene expression of APP and APLP2 at the transcriptional level at earlier disease timepoints should be considered to assess the functional implications of mRNA transcription during MND disease progression. These cumulative observations highlight how a lack of APLP2 expression in females is beneficial and this may be exerting a partial protective effect on the development of neurodegenerative disease associated with the spinal cord and with changes that can occur in ageing or in response to an oxidative stress response. Together, the effects of APLP2 on the pathological process of MND highlight a clear sex-dependent role.

Sex differences in human disease manifestations, progression and prevalence have been well noted in numerous illnesses including autoimmune disease [83], cardiovascular disease [84], cancers [85] and neurological disorders [86]. Earlier studies reporting sex differences in MND with a slightly higher prevalence in men compared to women (1.5:1, male to female ratio) [5, 10] were later found to be supported by a dose–response meta-analysis which showed that this sex-ratio decreased slightly with respect to population ageing [87]. This increased incidence in the male population is in line with the majority of epidemiological studies showing male MND patients tend to show an earlier age of disease onset [87], an effect that is also recapitulated in selective transgenic mouse models of MND [88, 89]. Although sex-specific differences have been identified in both the SOD1-G37R [90] and the SOD1-G93A mouse models for MND [15, 16, 18], our new findings identified sex-dependent effect associated with APP and APLP2 expression levels which may be linked to their neuroprotective properties in neurodegenerative disorders. Nevertheless, further clinical studies are needed to clarify the specifics of these sex differences involving SOD1 in MND and how APP and APLP2 contributes to this.

Another factor of paramount importance for the sex differences is the linked between APLP2 genes and sex hormones. Therefore, to further elucidate sex differences in the disease phenotype of the SOD1-G37R mouse lacking APLP2, sequence analysis of transcription factors in the promoter region of the mouse and human APLP2 gene was performed using the online transcription factor prediction program PROMO. We found multiple occurrences of the consensus sequence for transcription factor binding sites for progesterone, oestrogen and androgen on both the mouse and human APLP2 genes (Figure S7). This analysis is supported by the earlier report illustrating how the oestrogen receptor alpha (ERα), a key target for oestrogen and most active in females, played a key role in the regulation of the mitochondrial protein homeostasis (proteostasis) response in the SOD1-G93A mouse model for MND, an effect that was also sex dependent [91]. Indeed, a hallmark of MND is the accumulation of protein inclusions associated with perturbation on the efficiency of protein folding and protein degradation and together, this leads to the disruption of the intracellular proteostasis network of motor neurons and ultimately, to its demise [92]. Here, we present evidence of an increase in the hSOD1 expression in the female cohorts, however this was only significant in the female SOD1:APLP2 ± mouse group which further supports the sex-dependent linked of APLP2 expression in the protein accumulation of hSOD1 (Figure S4C), suggesting the possible involvement of APLP2 in SOD1-medicated pathogenic mechanism. Apart from abundant SOD1 aggregates the TAR-DNA binding protein (TDP-43) has been identified as the major component of the cytoplasmic inclusions in degenerating motor neurons [93]. The aggregation of these inclusions is regulated by phosphorylation and both autophagy and proteasome-mediated degradation pathways where they were observed to colocalize with autophagy markers p62 [94, 95]. As such, further research is warranted to determine whether the expression of APLP2 contributes proteostasis network of MND pathogenesis in a sex-dependent manner.

Other sex-dependent differences in MND have been linked to peroxisome proliferator-activated receptor coactivator 1-alpha (PGC-1α), a master regulator of mitochondrial metabolism [96, 97]. PGC-1α is a coactivator of ERα and regulates or enhances the transcriptional activity and signalling of androgen and oestrogen receptors [98, 99]. Since ablating PGC-1α gene expression in the SOD1-G93A mouse model for MND causes sex-dependent shortened survival time in male but not female [100], this supports the possibility that the sex differences we observed in the SOD1-G37R are mediated by these sex hormones through there interaction with APLP2 gene. However, whilst this idea is plausible, whether there is a unique interaction of APLP2 gene promoter region with the sex-dependent regulatory elements requires future experimental validation. In addition, whether this is linked to mitochondrial metabolism and PGC-1α is another avenue worth exploring in the future too. Nevertheless, our data provide a novel pathway linking APLP2 expression with sex-dependent effects in MND and this may explain the sexual dimorphism seen in MND patients. Whilst questions regarding the overlapping but distinct, functional roles of APP and APLP2 in MND remain unanswered, it is clear that APP and APLP2 can modulate the pathophysiology of MND in a sex-dependent manner.

## Conclusion

Our study indicates a role for APLP2 (and APP) in sex-dependent differences in MND. Whether this opens the prospect for a female-specific intervention for MND by targeting APLP2 function and/or expression remains unclear. A better understanding of the sex-specific functions of APLP2 (and APP) in MND identifies novel ways to modulate MND disease and this highlights the necessity to consider the role of sex differences in the development of MND therapies.

### Supplementary Information

Below is the link to the electronic supplementary material.

Supplementary file1 (DOCX 19693 KB)

## Data Availability

All data sets generated and analysed to support the conclusion of this study is included in this published article and its additional files.

## Abbreviations

AD: Alzheimer’s disease
APP: Amyloid precursor protein
APLP1: Amyloid precursor-like protein 1
APLP2: Amyloid precursor-like protein 2
ALS: Amyotrophic lateral sclerosis
CNS: Central nervous system
MND: Motor neurone disease
PNS: Peripheral nervous system
