# Supplementary material for: Sex-dependent effects of amyloid precursor-like protein 2 in the SOD1-G37R transgenic mouse model of MND
Source: Cell Mol Life Sci. 2021 Sep 2;78(19-20):6605–30. doi: 10.1007/s00018-021-03924-5 (PMC8558206; doi:10.1007/s00018-021-03924-5)
Supplement: Supplementary file 1 — Supplementary file1 (DOCX 19693 KB) [file 18_2021_3924_MOESM1_ESM.docx]

# Sex dependent effects of amyloid precursor-like protein 2 in the SOD1-G37R transgenic mouse model of MND.

Phan H. Truong^1,#,^*, Peter J. Crouch^1^, James B.W. Hilton^1^, Catriona A. McLean^2^, Roberto Cappai^1^ and Giuseppe D. Ciccotosto^1^

**Supplementary Methods**

**Immunofluorescence staining of spinal cord sections**

Serological transverse tissue sections (1:10) of the lumbar spinal cord were cut at 20 µm thickness (serially) using a cryostat machine (Leica) and were collected and adhered to superfrost plus slides (Fisher Scientific). Immunohistochemistry was prepared by briefly washing sections with PBS buffer three times (5 min per wash), permeabilised (0.3% Triton X in block buffer for 20 min) and blocked (10% goat serum in PBS) for 1 hour. Tissue sections were incubated with primary antibodies to anti-APLP2 95/11 (1:50), APP (22C11, 1:50) and anti-ChAT (Invitrogen, 1:500) diluted in blocking buffer overnight at 4°C in a humidifier chamber. The next day, antibodies were removed and slides washed with PBS buffer (three times, 10 min each) before incubation in a fluoro-tagged secondary antibody (goat anti-rabbit goat anti-mouse, and chicken anti-goat) prepared in blocking buffer and containing DAPI (2 µg/mL) for 1 hour at room temperature and in the dark. All tissue sections were washed with PBS (three times, 10 min each) and then covered with antifade mounting medium (Prolong Gold, Invitrogen) and mounted with a glass coverslip. The slides were air-dried at room temperature for at least 24 hours in the dark before imaging. The stained tissue sections were visualised through a 20X objective using a Zeiss Axioplan 2 microscope and images taken with a Coolscope snap camera and Zen 2 software (Zeiss, Germany).

**Supplementary Figure**

**
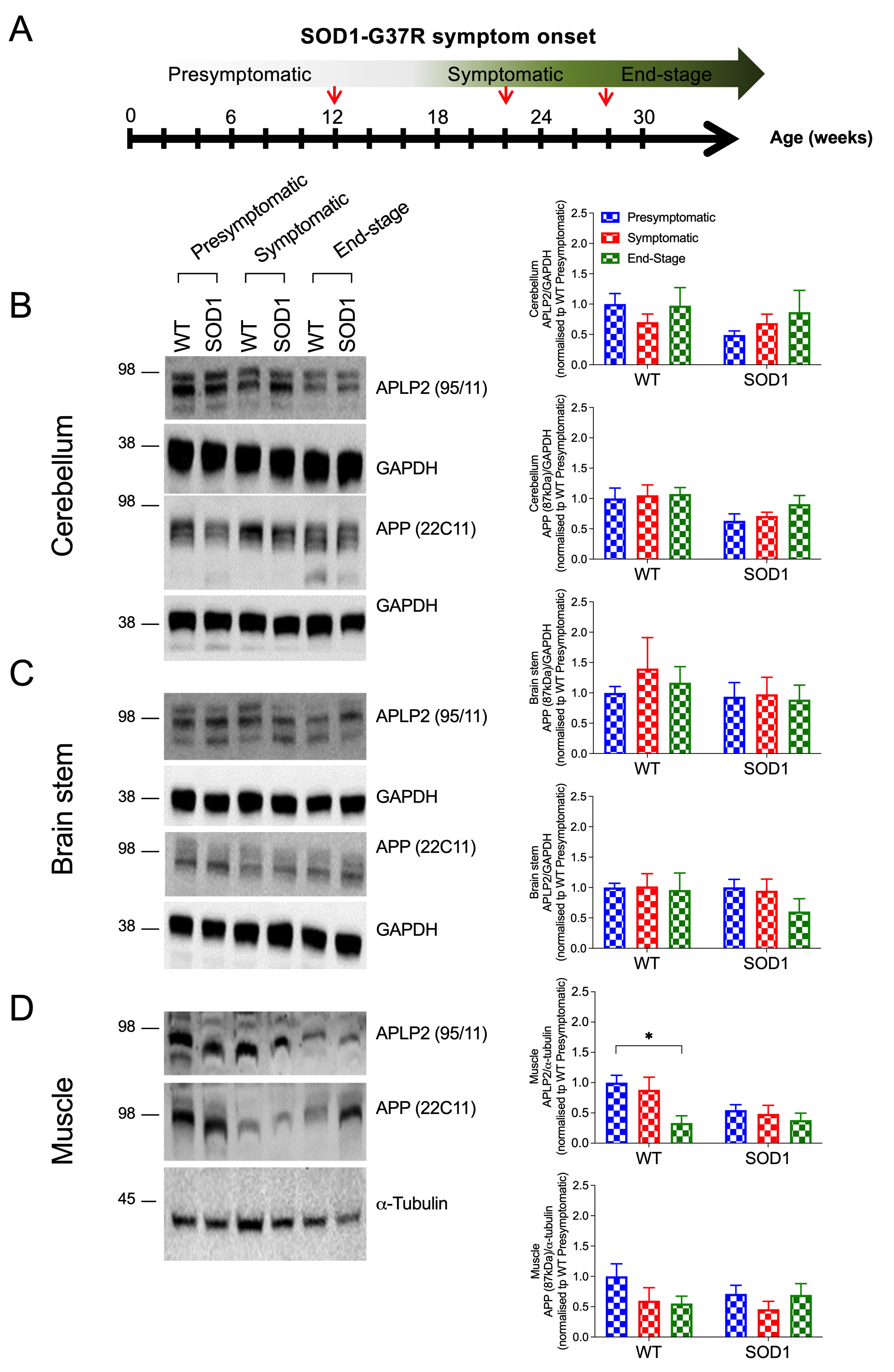
**

**Figure S1. (A)** Schematic representation of disease timeline of SOD1G37R mice. Immunoblot images and densitometric quantification of **(B)** cerebellum **(C)** brain stem and **(D)** muscle lysates for APP and APLP2 protein expression levels of SOD1-G37R mice and age-matched WT littermates at 12 weeks (pre-symptomatic), 22 weeks (symptomatic) and ~28 weeks (end-stage) old mice. Data are normalised to loading control protein (GAPDH) and expressed as a ratio of the WT pre-symptomatic sample for the respective tissue samples. Data present as mean ± SEM. Statistical testing by two-way ANOVA with Bonferroni’s post-hoc test, *p<0.05. n=4-6.

**
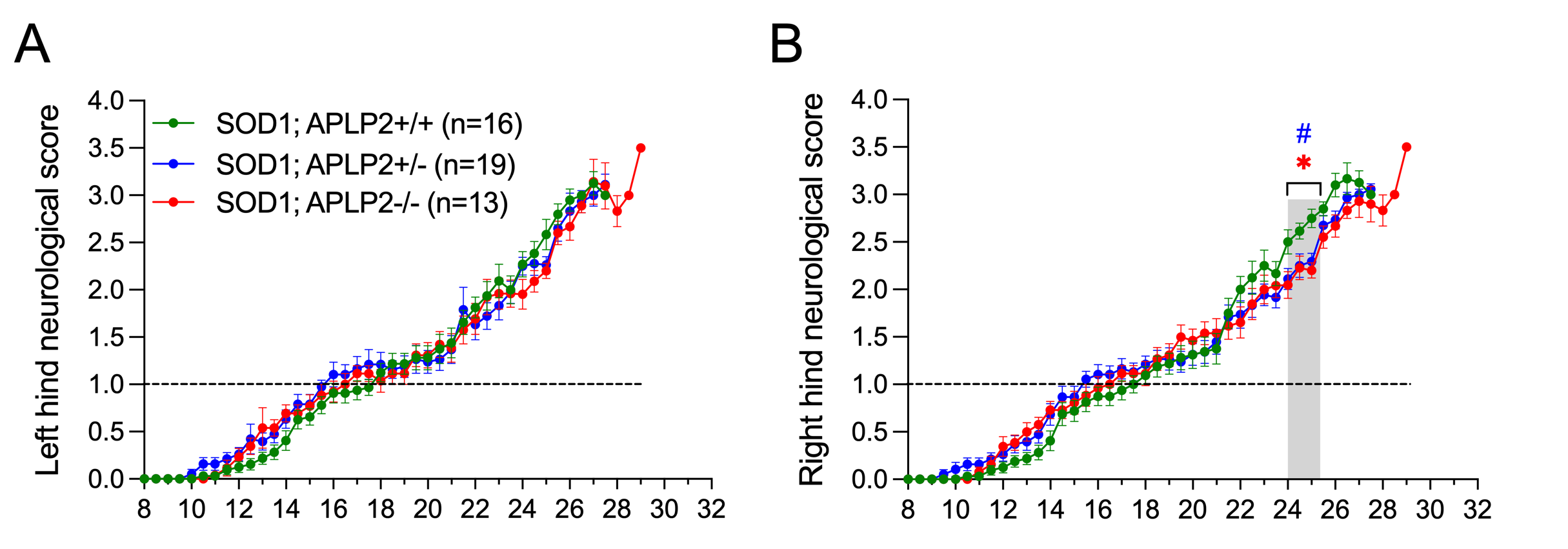
**

**Figure S2.** Neurological function scores for **(A)** left and **(B)** right hindlimbs for male groups SOD1-G37R:APLP2+/+, SOD1-G37R:APLP2-/- and SOD1-G37R:APLP2+/- mice from 8 weeks of age until disease End-stage.

**
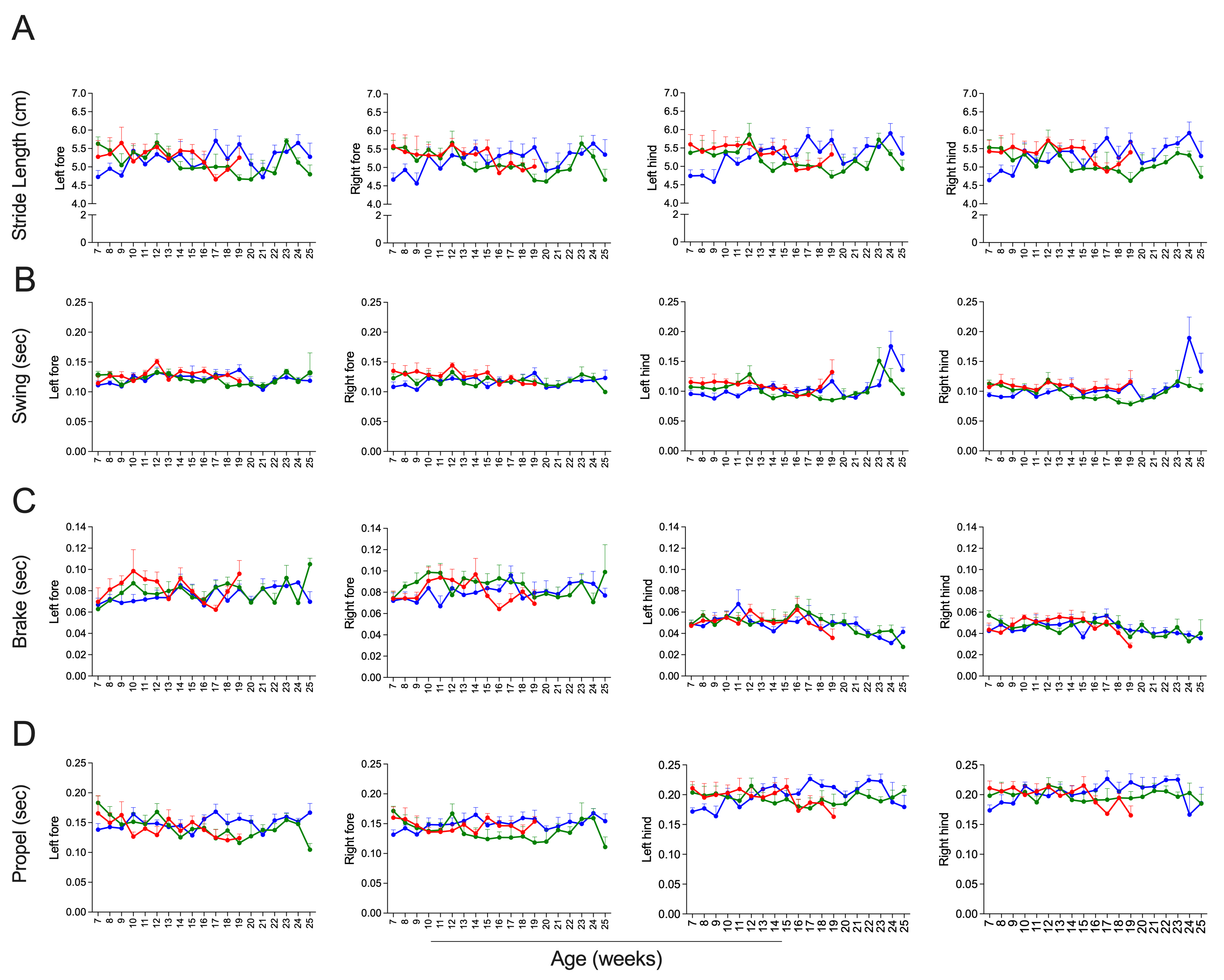
**

**Figure S3.** DigiGait analysis of left and right forelimbs and hindlimbs for **(A)** stride length (cm), **(B)** swing (seconds), **(C)** brake (sec) and **(D)** propel (seconds) of male SOD1;APLP2+/+, SOD1;APLP2-/- and SOD1;APLP2+/- mice from 7 -25 weeks of age at 15cm/s. Values presented as mean ± SEM statistical testing by compared to SOD1-G37R:APLP2+/+ using two-way ANOVA with Tukey’s post-hoc test, *p<0.05, #p<0.05. n=5-8.


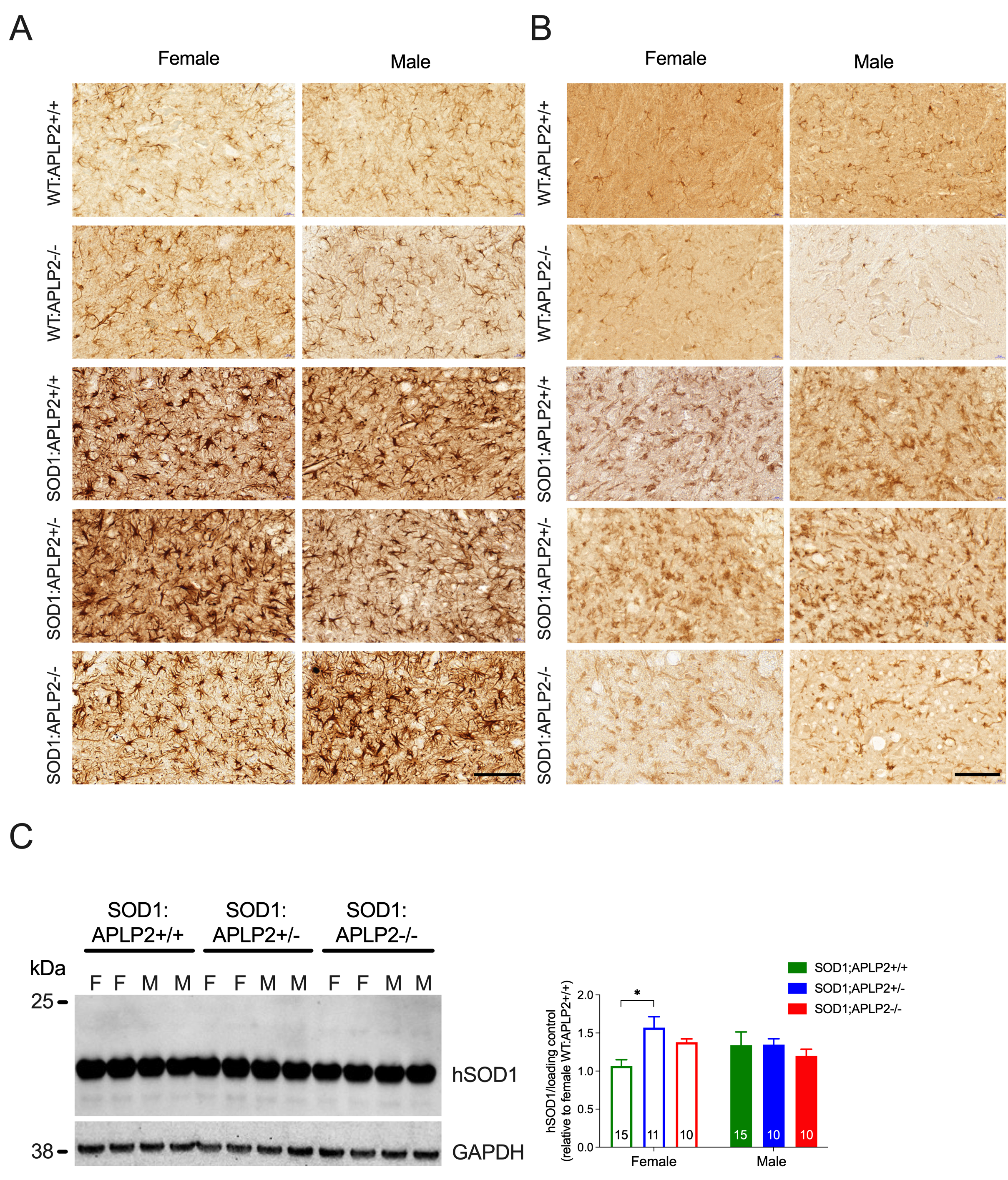


**Figure S4.** Representative immunohistochemical images and quantitative analysis of the ventral horn lumbar spinal cord and immunolabelled with **(A)** anti-GFAP and **(B)** anti-IBA1 of WT:APLP2+/+, WT:APLP2-/-, SOD1-G37R:APLP2+/+, SOD1-G37R:APLP2+/- and SOD1-G37R:APLP2-/- mice at disease end-stage. Scale bar = 100μm**. (C)** Representative immunoblots and densitometric quantification of hSOD1 in the spinal cord of SOD1-G37R:APLP2+/+, SOD1-G37R:APLP2+/- and SOD1-G37R:APLP2-/- mice at disease end-stage Values presented as mean ± SEM statistical testing by compared to SOD1-G37R:APLP2+/+ using two-way ANOVA with Tukey’s post-hoc test, *p<0.05, n=10-15.

**
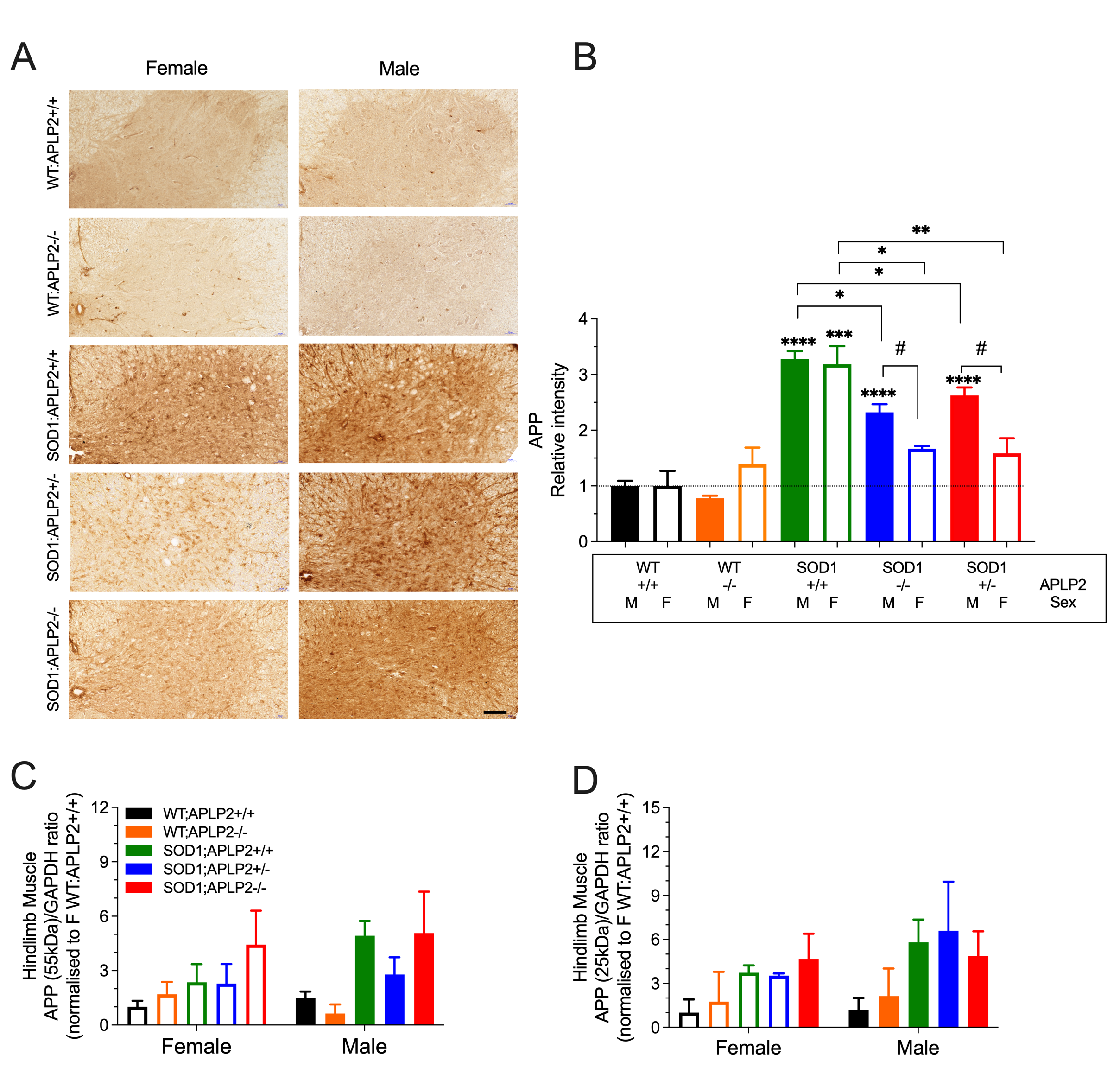
**

**Figure S5. (A)** Representative immunohistochemical images and (B) quantification of the relative pixel intensity of APP expression levels ventral horn lumbar spinal cord, immunolabelled with anti-APP (22C11) of the female and male groups SOD1-G37R:APLP2+/+, SOD1-G37R:APLP2-/-, SOD1-G37R:APLP2+/-mice at end stage of disease and their age-matched WT:APLP2+/+ and WT:APLP2-/- littermates control. APP levels were normalised to their respective male and female WT:APLP2+/+ mice groups respectively. Scale bar = 100μm. Densitometric quantification of APP hindlimb muscles detected at **(C)** 55kDa and **(D)** 25kDa for SOD1-G37R:APLP2+/+, SOD1-G37R:APLP2+/-, SOD1-G37R:APLP2-/- mice at disease end stage and WT:APLP2+/+, WT:APLP2-/- age-matched littermate controls. Values presented as mean ± SEM and statistical testing by one-way ANOVA with Bonferroni’s post-hoc test were used for genotype comparison to respective female (F) and male (M) WT:APLP2+/+ mice groups and student t-test was used for comparison between sexes, *p<0.05, **p<0.01, ***p<0.001, ****p<0.0001. n=4-6.

**
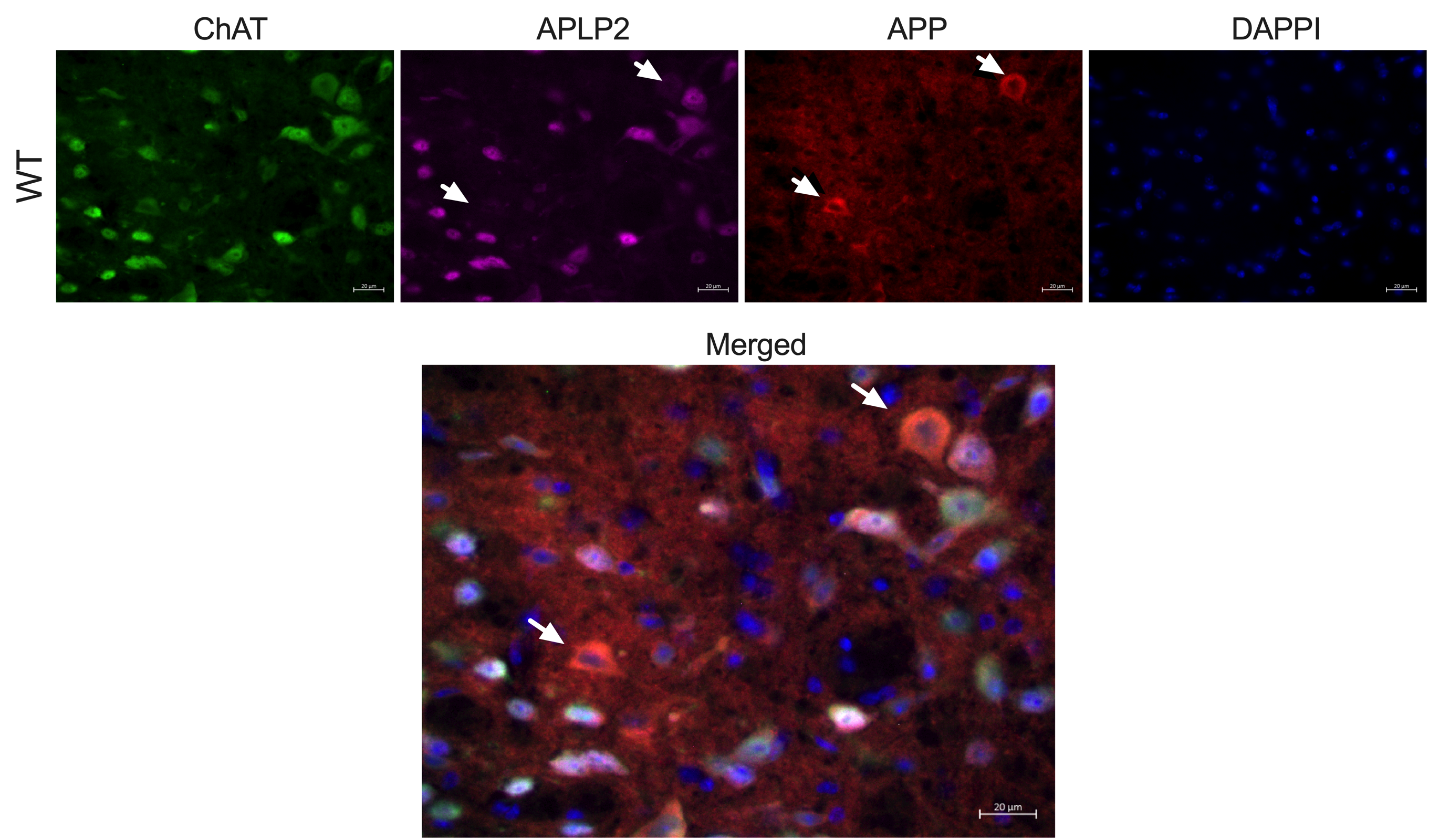
 Figure S6.** Immunofluorescence images of motor neurons in the ventral horn of the lumbar spinal cord of wild-type (WT) animals. Sections were triple labelled with anti-ChAT (green), DAPI (blue), ani-APP (red), and anti-APLP2 (magenta). Images indicated selectivity in a certain subgroup of motor neurons that are positively stained for ChAT colocalises with APP (arrowheads), and not APLP2 (arrowheads). Scale bar, 20µm.

**
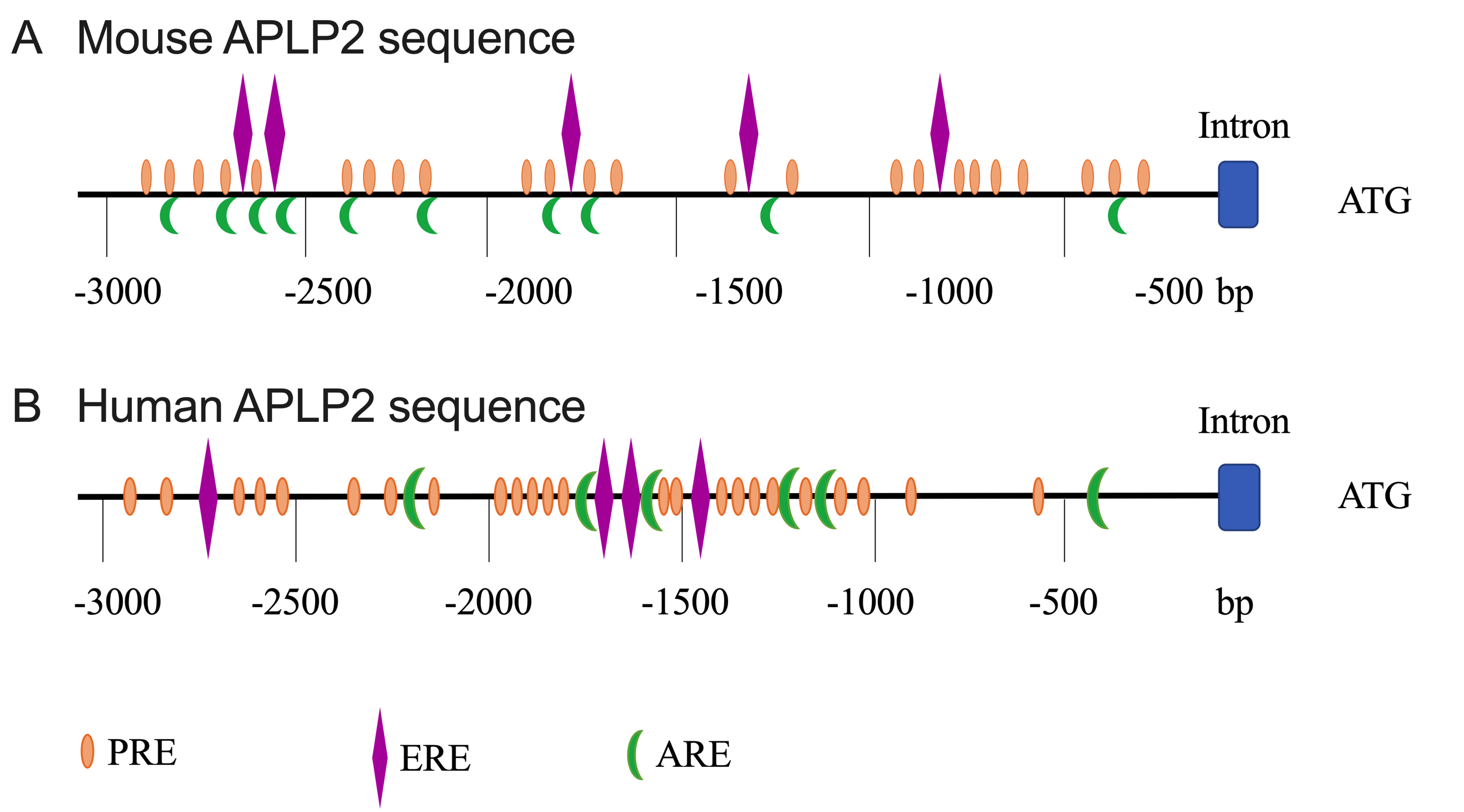
Figure S7.** Sequence analysis of mouse and human APLP2 promoter region shows the presence of sex hormone transcription factor binding sites. Schematic representation of APLP2 promoter region for (**A)** mouse and **(B)** human APLP2 gene. Shown are putative transcription factor binding sites along 3,000bp promoter length for progesterone response element (PRE) A & B, estrogen response element (ERE) alpha, and androgen response element (ARE).
